# The Genetic Basis of *Escherichia coli* Pathoadaptation to Macrophages

**DOI:** 10.1371/journal.ppat.1003802

**Published:** 2013-12-12

**Authors:** Migla Miskinyte, Ana Sousa, Ricardo S. Ramiro, Jorge A. Moura de Sousa, Jerzy Kotlinowski, Iris Caramalho, Sara Magalhães, Miguel P. Soares, Isabel Gordo

**Affiliations:** 1 Instituto Gulbenkian de Ciência, Oeiras, Portugal; 2 Unidade de Imunologia Clínica, Instituto de Medicina Molecular, Faculdade de Medicina da Universidade de Lisboa, Lisboa, Portugal; 3 Centro Biologia Ambiental, Faculdade de Ciências da Universidade de Lisboa, Lisboa, Portugal; Stanford University School of Medicine, United States of America

## Abstract

Antagonistic interactions are likely important driving forces of the evolutionary process underlying bacterial genome complexity and diversity. We hypothesized that the ability of evolved bacteria to escape specific components of host innate immunity, such as phagocytosis and killing by macrophages (MΦ), is a critical trait relevant in the acquisition of bacterial virulence. Here, we used a combination of experimental evolution, phenotypic characterization, genome sequencing and mathematical modeling to address how fast, and through how many adaptive steps, a commensal *Escherichia coli* (*E. coli*) acquire this virulence trait. We show that when maintained *in vitro* under the selective pressure of host MΦ commensal *E. coli* can evolve, in less than 500 generations, virulent clones that escape phagocytosis and MΦ killing *in vitro*, while increasing their pathogenicity *in vivo*, as assessed in mice. This pathoadaptive process is driven by a mechanism involving the insertion of a single transposable element into the promoter region of the *E. coli yrfF* gene. Moreover, transposition of the IS186 element into the promoter of *Lon* gene, encoding an ATP-dependent serine protease, is likely to accelerate this pathoadaptive process. Competition between clones carrying distinct beneficial mutations dominates the dynamics of the pathoadaptive process, as suggested from a mathematical model, which reproduces the observed experimental dynamics of *E. coli* evolution towards virulence. In conclusion, we reveal a molecular mechanism explaining how a specific component of host innate immunity can modulate microbial evolution towards pathogenicity.

## Author Summary

The selective pressure imposed by the host immune system is an important component of microbial adaptation from commensalism to pathogenicity. We used experimental evolution to study the initial steps of the adaptation of *Escherichia coli* to cells of the innate immune system, *i.e.*, macrophages. Our results demonstrate that bacteria can evolve remarkably fast, and acquire adaptations increasing survival inside macrophages and/or ability to escape engulfment. The mechanism underlying this pathoadaptive process involves the accumulation of mutations caused by transposon insertions, increasing pathogenicity *in vivo*. These findings reveal the remarkable fast pace at which bacteria can evolve to escape a central component of the host innate immunity, namely macrophages.

## Introduction

Bacteria can be used to study evolution in real time in controlled environments, *i.e.* experimental evolution [1]. Different studies have demonstrated that bacterial populations have an enormous potential to adapt to relatively simple abiotic challenges under laboratory environments [2], [3]. On the other hand, far less is known on how biotic interactions shape bacterial adaptive evolution. Antagonistic interactions (predation, parasitism) are likely to be important determinants of the rate of adaptive change observed in bacteria, their trait diversity and genome complexity [4], [5], [6]. The best-studied antagonistic interaction in an evolutionary laboratory setting is the one involving bacteria and their phages, which increases rates of bacterial adaptation and diversification [7], [8], demonstrating that biotic interactions can have an important role in bacterial evolution [9]. Another common antagonistic interaction faced by bacteria occurs when these infect mammals and are directly exposed to cells of the host immune system. To our knowledge this interaction has never been addressed in an experimental evolution context. Here, we determined the mechanisms via which *E. coli* evolve to overcome the antagonistic interaction imposed by one of the central components of host innate immunity, namely monocyte/macrophages (MΦ).

*E. coli* is both a commensal and a versatile pathogen, acting as a major cause of morbidity and mortality worldwide [10]. Moreover, there is evidence that some pathogenic *E. coli* evolved from commensal strains [11], [12], making *E. coli* an ideal organism to study the transition from commensalism to pathogenicity. *E. coli* colonizes the infant gastrointestinal tract within hours after birth, and typically builds a mutualistic relation. However, non-pathogenic strains of *E. coli* can become pathogenic, when the gastrointestinal barrier is disrupted as well as in immunosuppressed hosts [13], [14], [15].

MΦ are a key component of host defense mechanisms against pathogens [16]. They can provide direct bactericidal response through phagocytosis, a process by which bacteria are killed inside endocytic phagosomes, through the generation of reactive oxygen and nitrogen species among other effector mechanisms. Yet many bacterial species are capable to escape and resist eukaryotic cells [17], [18], suggesting that several bacterial defense mechanisms evolve upon encounter with MΦ. Adaptive microbial mechanisms to escape MΦ include surface masking and capsule formation (to avoid engulfment and phagocytosis), increased motility, filamentation and biofilm formation. Mechanisms acting after engulfment by MΦ include toxin release. Within the species of *E. coli* alone, there are examples of several different mechanisms [19].

In the present study, we established an *in vitro* system in which *E. coli* is allowed to evolve under continuous selective pressure of MΦ, and ask how quickly and by which mechanisms commensal *E. coli* evolve resistance to one of the sentinels of the innate immune system, the MΦ.

## Results

### Emergence of morphological diversity and dynamics of phenotypes

We followed the evolution of six *E. coli* populations (all founded from the same ancestral clone), when adapting to the antagonistic interaction imposed by the murine monocytic cell line (RAW 264.7), referred throughout the text as MΦ. The bacterial populations (M1 to M6) evolved, by serial passage, in complete culture medium with MΦ and were propagated at a multiplicity of infection (MOI) of 1∶1 (10^6^ *E. coli* to 10^6^ MΦ, see Fig. S1). After 24 hours bacterial numbers reach around 4×10^8^ and are subsequently bottlenecked to start the next passage with 10^6^ bacteria again. In parallel, we also evolved *E. coli* under identical experimental conditions in the absence of MΦ (the resulting evolved clones are named CON). In this case the population is propagated by daily passages involving a bottleneck of 10^4^ cells at each passage. This results in ∼15 generations per day, given the increase in bacteria numbers observed during 24 hours. All populations evolved for a period of 30 days, which corresponds to approximately 450 generations. We note that this is an approximate value because as adaptation proceeds the population dynamics will change and differences in the number of generations per day will occur.

**Figure 1:**
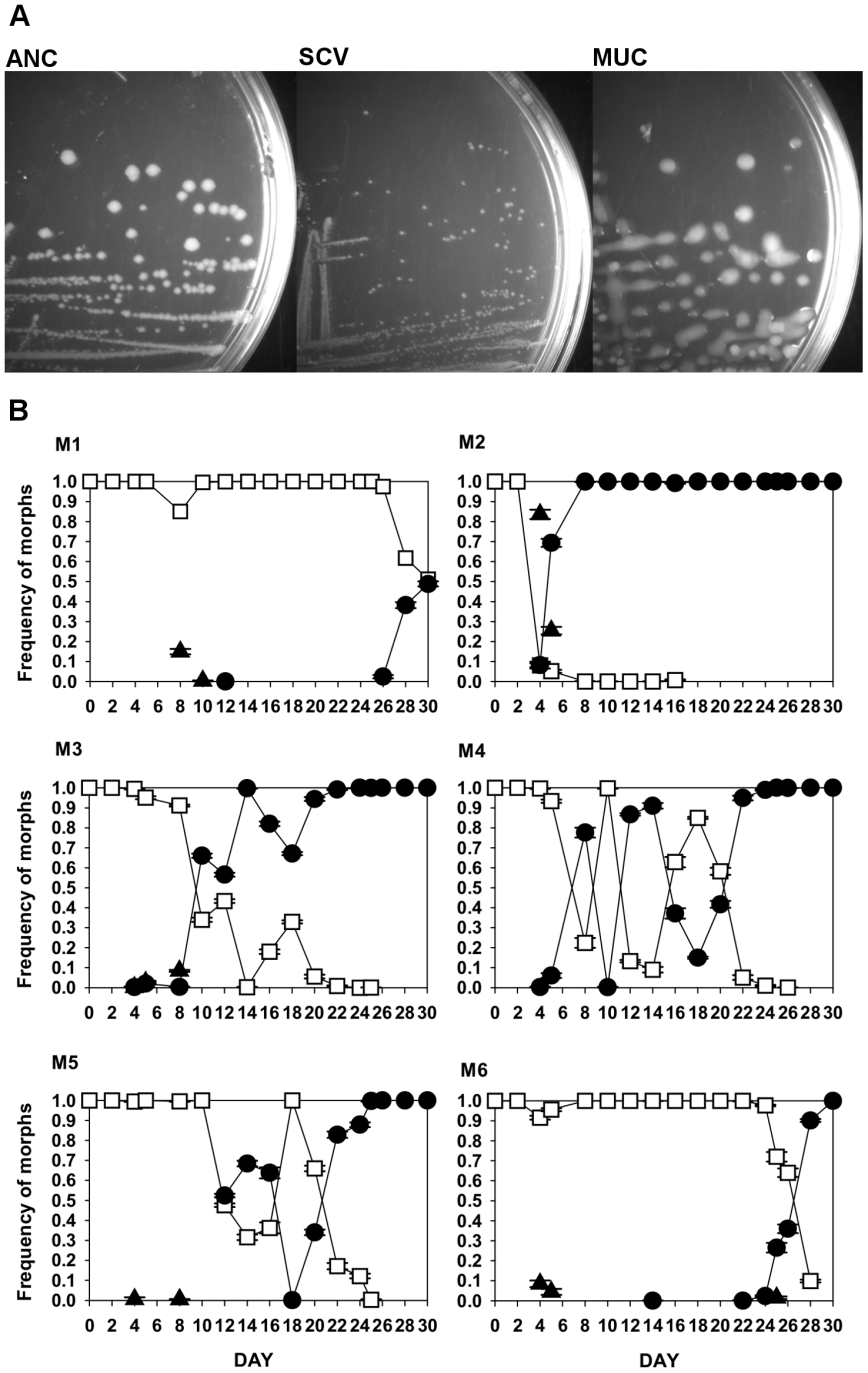
Emergency of morphological diversity in the bacterial populations adapting to MΦ. (A) Examples of the variability for colony morphology that emerged in *E. coli* populations adapting to MΦ, from left to right – ANC stands for morphology of ancestral, SCV for the small colony variants morphology and MUC for the mucoid colony morphology. (B) Dynamics of frequency change of the evolved phenotypes in each replicate evolving populations (M1 to M6): white squares indicate ANC, black triangles SCV, black circles MUC phenotypes.

Adaptation of the bacterial lines in the presence of MΦ was characterized by the emergence of phenotypic variation within populations. After 4 days of evolution, i.e. approximately 60 generations, distinct colony morphologies emerged in all populations, detected when plating on LB plates (Fig. 1A). Such morphological diversity was never observed in control populations evolved for 30 days under the same experimental conditions in the absence of MΦ (n = 6). Two distinct heritable morphs were identified and scored, *i.e.* small colony variants (SCV) and large translucid mucoid (MUC) colonies and their frequencies were quantified over time (Fig. 1B). SCVs were observed in five out of six populations, but this morph remained at low frequency and was only detected transiently. The parallel emergence of SCVs in independent evolving populations, suggests that this phenotype constitutes an initial adaptation of *E. coli* to the antagonistic interaction imposed *in vitro* by MΦ. In contrast, MUC clones which rose in frequency in all populations, reached fixation in five out of six populations by day 30. The changes in frequency of SCVs and MUCs showed complex dynamics (Fig. 1B). In some populations, once SCVs decreased in frequency MUCs tended to increase, *e.g.* populations M2 and M3. This suggests that MUCs can outcompete SCVs, presumably due to a larger fitness advantage. These observations suggest that *E. coli* morphological diversity can emerge very rapidly as a result of their adaptation to MΦ.

### Fitness increase and phenotypic characterization of SCV and MUC clones

**Figure 2:**
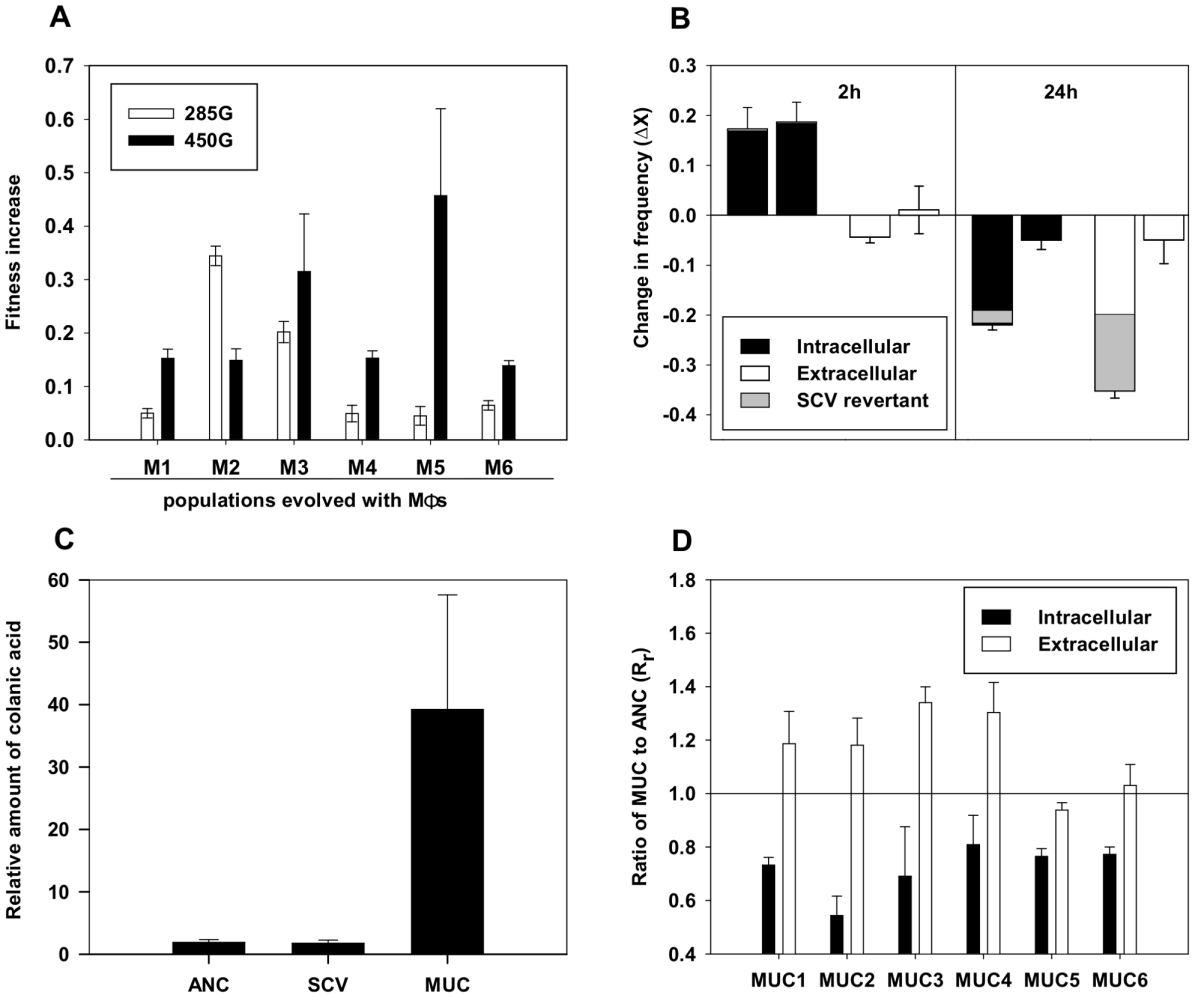
Phenotypic characterization of evolved populations. (A) Fitness increase of M1 to M6 populations relative to the ancestral clone at 285 (white bars) generations and 450 (black bars) generations. Error bars correspond to 2SE. (B) Competitive fitness of SCV clones in presence of MΦ. The change in frequency (ΔX) of the evolved bacteria against the ancestral in the intracellular (black bars) and extracellular (white bars) niche of the MΦ at MOI (1∶1). Clones are ranked in the following order: SCV_M1_D8 and SCV_M3_D5. Because SCV clones revert to ancestral looking colonies, frequencies of those phenotypic revertant SCV_REV colonies are shown in grey. (C) MUC clones overproduce colanic acid. After purification from the growth medium of each clone (SCV_M1_D8, MUC_M3_D19 and ANC), the amount of colanic acid was determined by measuring non-dialyzable methylpentose (fucose) absorbance at 396 and 427 nm after reaction with sulfuric acid and cysteine hydrochloride. Measurements were repeated three times for each clone. Obtained values (ΔA396–ΔA427) were directly correlated with fucose calibration curve (see Fig. S2) and normalized for CFUs. (D) Evidence that MUC clones adapted to better escape MΦ phagocytosis. Rr represents the relative abundance (R_r_) of evolved clones to that of the ancestral at 3 h of infection. Clones MUC1 to MUC6 were sampled from each independent evolution. In black bars the relative abundance inside MΦ and in white bars outside MΦ. All evolved clones show a smaller abundance inside MΦ, suggesting that these are better adapted to escape MΦ phagocytosis. Error bars correspond to 2SE.

Competitive fitness of *E. coli* populations was measured at two time points during the process of evolution (day 19∼285 generations and day 30∼450 generations), revealing that all populations exhibit a significant fitness increase (Fig. 2A). On average, fitness increase was of 0.10 (2SE = 0.07) and 0.27 (2SE = 0.10) after 19 and 30 days, respectively. Fitness increased between generations 285 and 450 across populations (Students' paired t-Test, P = 0.02).

The observation that SCVs emerged in at least 80% of the independent evolving populations but with low frequency strongly suggests that SCVs have a transient selective advantage that is outcompeted over time. To better understand this selective advantage we performed two assays: 1) exposure of MΦ *in vitro* to SCVs to test for possible intracellular versus extracellular growth differences relative to that of the ancestral strain; 2) a fitness assay to determine the ability of SCV to outcompete the ancestral strain, in the presence of MΦ. We did not observe any difference in SCV growth either intracellularly (Rr = 0.99+0.16 (2SE)) or extracellularly (Rr = 1.01+0.13 (2SE)) relative to the ancestral non-evolved clone, while there was an advantage in the competitive fitness assay (Fig. 2B). SCVs (clones SCV_M1_D8 and SCV_M3_D5) exhibited a fitness advantage relative to the ancestral strain, inside MΦ, as assayed 2 hours after infection. However, this advantage was restricted to the early phase of infection, given that SCVs showed a disadvantage outside MΦ 24 hours after infection (Fig. 2B). These results probably explain why SCVs increased in frequency but failed to reach fixation (see Fig. 1B).

We tested the *in vitro* evolved SCVs for traits common to those of clinical SCV isolates from different bacterial species [20] [21]. The evolved *E. coli* SCVs showed an increased resistance to aminoglycosides, but not to other antibiotics (see Supplemental Table S1), were catalase negative and showed a remarkable instability. In rich medium SCVs reverted to a large colony phenotype at a frequency of 9×10^−4^ (2SE = 4×10^−4^) and supplementation with hemin enhanced their growth relative to the ancestral (SCV_M1_D8: 2.9±1 (2SE) and SCV_M3_D5: 2.5±0.7(2SE)). These results imply that the selective pressure of MΦ led to the emergence of phenotypes typical of pathogenic bacteria.

Mucoidy, the trait evolved in the MUC clones, is also a trait observed in certain infections, for example in *Pseudomonas aeruginosa* or *E. coli* [22], [23]. The *in vitro* evolved MUCs produce high levels of exopolysaccharides when plated on LB. Since colanic acid is present in most natural *E. coli* isolates [24], and this capsule is made in mutants of *E. coli* that emerge under stress conditions [25], we tested mucoid clones for overproduction of this exopolysaccharide. Mucoid clones showed overproduction of colanic acid (Fig. 2C, Fig. S2), suggesting that rapid evolution to change this trait can occur under the specific selection pressure imposed by MΦ. We tested whether MUCs escaped MΦ engulfment, by quantifying the relative abundance of intracellular and extracellular of MUC after 3 hours co-incubation with MΦ. Relative abundance of intracellular bacteria in MΦ was lower for MUC versus the ancestral strain in 6 out of 6 MUC clones tested (Fig. 2D). Moreover, the extracellular abundance of MUC clones relative to ancestral was higher in 4 out of 6 MUCs tested. We then asked whether MUCs would trigger MΦ cytotoxicity, a process that would contribute to reduce the negative impact exerted by MΦ on MUC versus ancestral clones. MΦ cytotoxicity was similar in the presence of MUC versus ancestral clones (Fig. S3A and Fig. S3B). Furthermore MUCs did not cause any significant changes in MΦ ability to engulf the ANC clone (Fig. S3C). Taken together, these results strongly suggest that MUCs are better adapted to escape MΦ but do not diminish the ability of MΦ to internalize ancestral *E. coli*.

### *E. coli* evolved *in vitro* to escape MΦ show increased virulence *in vivo*

**Figure 3:**
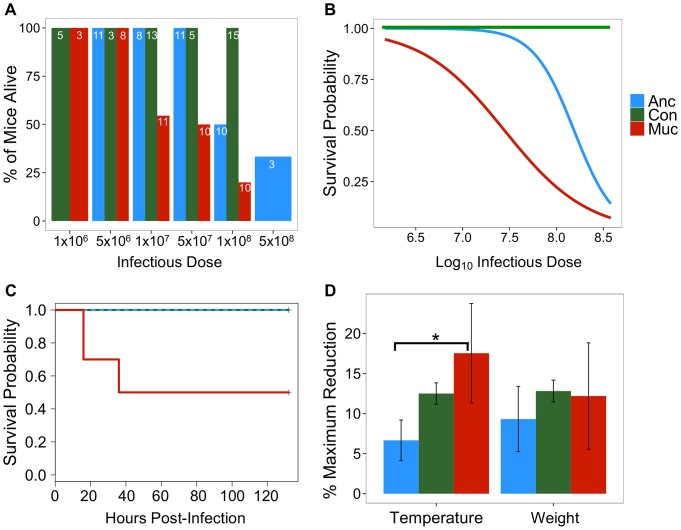
*In vitro* evolved *E. coli* show increased virulence *in vivo*. **A**) Survival of mice infected with different doses of ancestral (ANC, in blue), mucoid bacteria evolved in the presence of MΦ (MUC, in red) or bacteria evolved in the absence of MΦ (CON, in green). The number of mice are shown inside the bars, **B**) Survival probability of mice infected with ANC, MUC and CON, represented as lines from the fit of a binomial General Linear Model used to infer LD_50_, **C**) Kaplan-Meier curves and **D**) % maximum reduction in temperature or weight at the LD_50_ dose for the MUC (n = 10), ANC (n = 11) and CON (n = 5) (Error bars correspond to 2SE, * indicates p<0.05).

We tested whether adaptation of evolved MUC clones to escape MΦ is associated with increased virulence. We compared the survival of mice infected systemically via the intra-peritoneal route, with increasing amounts of MUC versus ANC bacteria or bacteria that evolved in the absence of MΦ (CON) (Fig. 3A). The lethal dose 50 (LD_50_) of MUC infection (LD_50_: 2.8×10^7^, with 95% CI 1.4×10^7^–5.8×10^7^) was 5–10 times lower than that of ANC (LD_50_: 1.6×10^8^, with 95% CI 8.5×10^7^–2.8×10^8^) or CON (LD_50_: above 1×10^8^), as inferred from the confidence intervals (Fig. 3A and 3B), suggesting that MUC clones have increased virulence. In agreement with these observations, infection with ancestral or with bacteria evolved in the absence of MΦ at a dosage corresponding to the MUC LD_50_ was not lethal, i.e., 100% survival of mice occurred (log-rank test: χ^2^_2_ = 9.9, *p* = 0.007; Fig. 3C). Higher lethality of MUC infection was associated with significant reduction in temperature (but not weight), as compared to infection with ANC bacteria at the dosage corresponding to the MUC LD_50_ (χ^2^_2_ = 0.61, *p* = 0.0004; Fig. 3D).

We then asked whether MUC bacteria elicited a MΦ response *in vitro* that would be somehow altered, as compared to the response elicited under the same conditions by the ANC or CON clones. When co-cultured with MUC, primary mouse peritoneal MΦ produced similar levels of the pro-inflammatory cytokine TNF, as compared to MΦ co-culture with ANC or CON clones (see Text S1 and Fig. S4). This suggest that although MUC clones have evolved to escape MΦ *in vitro* and increasing their pathogenicity *in vivo*, these clones are still readily detected by MΦ, as revealed by TNF secretion. This read out was used hereby as out-put of pattern recognition receptor triggered signaling leading to the activation of a core pro-inflammatory signal transduction pathway, which appears to be equally responsive to the different bacterial clones tested.

Overall our results show that the MUC clones, which overproduce colanic acid and dominated the bacteria populations during the interaction with MΦ, exhibit increased virulence.

### Genetic basis of the adaptation to macrophages

**Figure 4:**
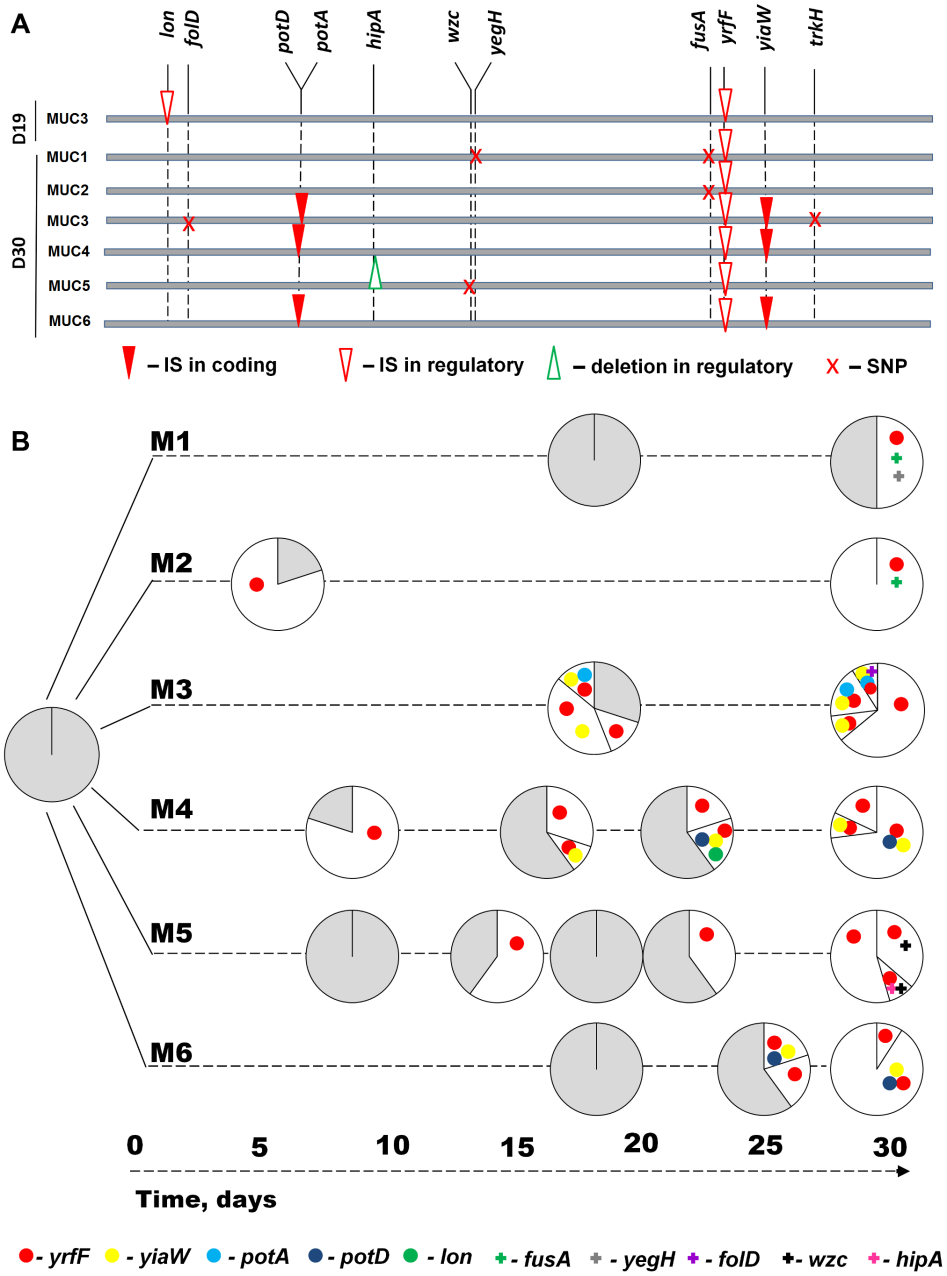
Genetic characterization of adaptive mutations and the dynamics of their appearance. (A) Mutations identified in MUC1 to MUC6 clones isolated from M1 to M6 populations (evolved for 450 generations), represented along the *E. coli* chromosome. For simplicity, the genomes are represented linearly and are horizontally drawn. The types of mutations are represented in the following way: SNPs are shown as crosses, IS insertions as inverted triangles and deletions as triangles. Filled symbols represent mutation in the coding region of the gene and empty symbols in the regulatory region. (B) Emergence and spread of adaptive mutations in M1 to M6 populations. Dynamics of haplotype frequencies in evolving populations at different days of evolution experiment are represented by circles. The color and symbol (IS insertions are represented as circles and other mutations as crosses) of each sector represents different haplotypes and the area of the circle their frequency in the population. Grey area represents the frequency of clones in the population that were typed for existing mutations in the population and did not differ from ancestral haplotype.

Given the phenotypes of the MUCs and their dynamics, we sought to determine the molecular basis of the mutations responsible for their increase in frequency along the evolutionary process. Whole genome sequencing of a clone sampled from M3 population at day 19 of the evolution process (MUC_M3_D19) revealed that it carries two transposon insertions, *i.e.* a IS186 insertion into the promoter region of *lon* and one IS1 insertion upstream of the *yrfF* gene (see Table 1). The IS1 insertion event occurred in all sequenced clones sampled at day 30 (Table 1, Fig. 4A), revealing parallelism at the genetic level across all independently evolved lines. The function of the *yrfF* gene is unknown in *E. coli*, but its homologue in Salmonella, *i.e. igaA*, prevents over-activation of the Rcs regulatory system, which regulates colanic acid capsule synthesis [26]. It is therefore likely that the insertion upstream of *yrfF* alters *E. coli* ability to produce colanic acid, in keeping with the observation that MUC clones produce high levels of colanic acid, as compared to ANC bacteria (Fig. 2C).

**Table 1:** Mutations acquired by evolved clones identified through whole genome re-sequencing (WGS).

Clone	Genome Position	Gene	Mutation	Annotation
**MUC_M3_D19**	360771	*clpX/lon*	intergenic (+88/−100)	IS186 +12 bp
	3411601	*nudE/yrfF*	intergenic (−273/−47)	IS1 +10
**MUC1**	2029672	*yegH*	G→T	A422S GCC→TCC
	3356932	*fusA*	A→C	S588A TCC→GCC
	3411601	*nudE/yrfF*	intergenic (−273/−47)	IS1 +10
**MUC2**	3356932	*fusA*	A→C	S588A TCC→GCC
	3411605	*nudE/yrfF*	intergenic (−277/−43)	IS1 +6
**MUC3**	459734	*folD/sfmA*	G→T	intergenic (−10/−461)
	1088154	*potA*	coding (589/1137 nt)	IS1 +10
	3411601	*nudE/yrfF*	intergenic (−273/−47)	IS1 +10
	3640515	*yiaW*	coding (263/324 nt)	IS1 +9
	3922002	*trkH*	T→A	L389Q CTG→CAG
**MUC4**	1084946	*potD*	coding (1032/1047 nt)	IS1 +9
	3411601	*nudE/yrfF*	intergenic (−273/−47)	IS1 +10
	3640515	*yiaW*	coding (263/324 nt)	IS1 +9
**MUC5**	1480525	*ydeS/hipA*	intergenic (−1603/+205)	Δ208 bp
	2024227	*wzc*	G→T	P645T CCG→ACG
	3411601	*nudE/yrfF*	intergenic (−273/−47)	IS1 +10
**MUC6**	1084946	*potD*	coding (1032/1047 nt)	IS1 +9
	3411601	*nudE/yrfF*	intergenic (−273/−47)	IS1 +10
	3640515	*yiaW*	coding (263/324 nt)	IS1 +9

Mutations in intergenic regions have the two flanking genes listed (e.g., *clpX*/*lon*). SNPs are represented by an arrow between the ancestral and the evolved nucleotide. Whenever a SNP gives rise to a non-synonymous mutation the amino acid replacement is also indicated. The symbol Δ means a deletion. For intergenic mutations, the numbers in the Mutation row represent nucleotides relative to each of the neighboring genes, here + indicates the distance downstream of the stop codon of a gene and − indicates the distance upstream of the gene, that is relative to the start codon. Insertions of IS elements are denoted by the specific IS element followed by the number of repeated bases caused by its insertion.

Other important parallelisms (observed in 3 out of 6 populations analyzed) include two transposition events, namely, one in *yiaW* coding region and the other in the coding region of the *pot* operon. *potD* is one of the four genes of the *potABCD* operon, a spermidine-preferential uptake system [27]. All four genes are essential for spermidine uptake, indicating that the insertions in *potD* detected in clones MUC_M4_D30 and MUC_M6_D30, or the insertion in *potA* observed in clone MUC_M3_D30, are likely to impair uptake of spermidine. We tested the effect of polyamines in the evolved MUC clones and observed that while all exhibit a growth advantage in the presence of spermidine, the clones with insertions in *potD* (MUC_M4_D30 and MUC_M6_D30) exhibit an increased growth advantage compared to the other MUC and the ancestral, in the presence of spermine (Fig. S5). During adaptation to MΦ, insertion in *yiaW* (whose function is unknown) was followed rapidly by insertion in *potA* or *potD* genes (see Fig. 4B, M3, M4 and M6 populations), indicating a potential interaction between these two events. This parallelism was observed in populations exposed to MΦ and not in bacteria that evolved in the absence of MΦ, suggesting that insertions in *yiaW* contribute functionally to adaptation of *E. coli* to MΦ. Given that many of the adaptive mutations observed under the different forms of stress imposed by MΦ were caused by IS insertions, we tested if the frequency of spontaneous mutations (including IS insertions) is higher in the presence versus absence of MΦ in the ancestral strain. No significant differences were found, suggesting that selection was the main force driving the increase in frequency of IS elements (see Text S1 and Fig. S6).

Other parallelisms were observed at the level of point mutations in two clones with the same non-synonymous SNP in *fusA*, a gene coding for elongation factor G, which catalyzes the elongation and recycling phases of translation [28]. Mutations in *fusA* reduce the rate of protein synthesis, a hallmark of stress responses, with pleiotropic effects on bacterial physiology [29]. Mutations in *fusA* have also been related with the development of SCVs in *S. aureus* [30]. We sequenced *fusA* in our *in vitro* evolved *E. coli* SCVs (11 clones sampled from M1 population at day 8 and 10 clones sampled at day 4) but did not find any substitutions in this gene.

### Dynamics of haplotypes and emergence of a transient mutator

**Figure 5:**
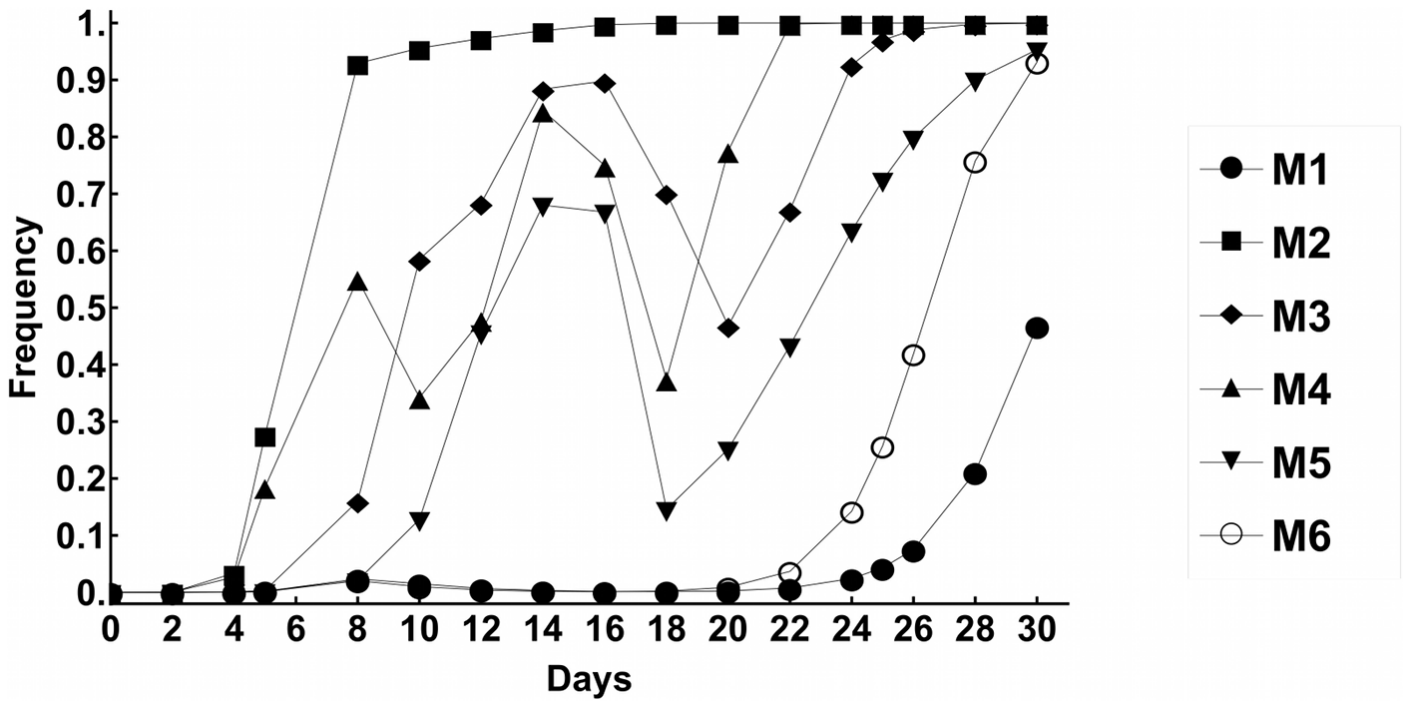
Predictions of model of clonal interference for changes in mucoid frequencies with time. Simulations of the adaptive dynamics over the period of the experiment (30 days). The frequencies of mucoid phenotypes are plotted and can be compared to those observed in the experiments (Fig. 1B). The values of parameters used and the dynamics of haplotypes that compete for fixation are shown in Figure S9.

To further understand the dynamics of adaptation in each independent evolved bacterial population, we sought to determine the frequency of the mutations found (see Table 2), in clones sampled along the evolution experiment. Adaptation involved the competition between distinct haplotypes and the successive accumulation of beneficial mutations, mainly caused by IS insertions (Fig. 4B). Such haplotype dynamics is characteristic of clonal interference [3], where clones carrying distinct beneficial mutations compete for fixation. We modeled this process, within the basic ecological scenario of our experiment (see Fig. 5 and Text S1, Fig. S7 to S11), fully reproducing the complex dynamics of the mucoid and non-mucoid phenotypes observed in Figure 1B.

**Table 2:** Clones that were typed for existing mutations in M1 to M6 populations.

Population	Day	Morph	Nr. of clones	*yrfF*	*lon*	*fusA*	*yegh*	*yiaW*	*potA*	*PotD*	*folD*	*trkH*	*hipA*	*wzc*
**M1**	18	ANC	10	−	−	−	−							
	30	MUC	11	+	−	+	+							
	30	ANC	10	−	−	−	−	−	−	−				
**M2**	5	MUC	8	+	−	−								
	5	ANC	2	−	−	−								
	30	MUC	11	+	−	+		−	−	−				
**M3**	18	ANC	10	−	−			−	−		−	−		
	18	MUC	6	+	−			+	−		−	−		
	18	MUC	2	+	−			+	−		−	−		
	18	MUC	2	+	−			−	−		−	−		
	30	MUC	7	+	−			−	−		−	−		
	30	MUC	2	+	−			+	+		−	+		
	30	MUC	1	+	−			+	−		−	+		
	30	MUC	1	+	−			+	+		+	+		
**M4**	8	MUC	8	+	−			−		−				
	8	ANC	2	−	−			−		−				
	16	ANC	6	−	−			−		−				
	16	MUC	3	+	−			−		−				
	16	MUC	1	+	−			+		−				
	20	ANC	6		−			−		−				
	20	MUC	2	+	+			+		+				
	20	MUC	2	+	−			−		−				
	30	MUC	2	+	−	−		−		−				
	30	MUC	1	+	−	−		+		−				
	30	MUC	8	+	−	−		+		+				
**M5**	8	ANC	10	−	−								−	−
	14	ANC	4	−	−								−	−
	14	MUC	6	+	−								−	−
	18	ANC	10	−	−								−	−
	20	ANC	6	−	−								−	−
	20	MUC	4	+	−								−	−
	30	MUC	1	+	−	−		−	−	−			+	+
	30	MUC	5	+	−	−		−	−	−			−	+
	30	MUC	5	+	−	−		−	−	−			−	−
**M6**	18	ANC	10	−	−			−		−				
	26	ANC	6	−	−			−		−				
	26	MUC	2	−	−			−		−				
	26	MUC	2	+	−			+		+				
	30	MUC	1	+	−	−		−		−				
	30	MUC	10	+	−	−		+		+				

An IS186 insertion into the promoter region of *lon*, was observed in clones sampled from populations M3 and M4 (Fig. 4). Lon (Long Form Filament) is a heat shock protease responsible for degradation of defective proteins in the cell [31]. The promoter region of *lon* is a hotspot for IS186 insertions [31], which may contribute to the occurrence of this mutation in independently evolved clones. We tested if the proportion of spontaneous *lon*::IS186 mutants is higher in the presence versus absence of MΦ, however no difference was observed (see Text S1). As *lon* mutants tend to overproduce colanic acid [32], a trait that appears to be strongly selected for in our experimental system, it is possible that this was the main beneficial effect caused by the insertion. However, the IS186 insertion could only be detected at intermediate time points in the experiment and not at day 30 (see Fig. 4B). Interestingly, *lon* has been reported to be a mutator gene in mutants that bear an IS186 insertion in its promoter, thus increasing the rate of IS transpositions 10- to 100-fold [33]. This happens because the stability of several transposases is dependent on the Lon protease [34], [35], which seems to regulate their transposition activity. We tested MUC_M3_D19 for increased mutagenesis. This clone carries an IS186 inserted in −10 promoter region of *lon* and since mutations in this position were shown to significantly decrease level of *lon* transcription [36], it is likely that it could be a mutator. If so this could contribute to the burst of transposition events that occurred during adaptation. We found a significant increase in the frequency of D-cycloserine resistant clones in MUC_M3_D19 relative to the ancestral non-evolved clone (median frequency 2.6×10^−6^ vs. 1×10^−7^, for the ancestral background, P = 5.5×10^−13^, W = 203.5, Mann-Whitney U test, Fig. S12). Consistent with this increased mutagenesis being driven by IS insertions, no significant differences in the frequency of rifampicin resistant clones, which are caused by point mutations, were observed (median frequency 3.3×10^−7^ vs 6.9×10^−7^ for the ancestral background, P = 0.1, W = 21, Mann-Whitney U test). The presence of IS186 in the *lon* promoter region was also found to be highly unstable. A spontaneously derived non-mucoid clone from MUC_M3_D19 (MUC_M3_D19_REV) shows a precise excision of this element, while maintaining the IS1 insertion in regulatory region of *yrfF* (see Table 1). These results indicate that this IS186 insertion enhances mucoidy levels, increases mutagenesis and is also very unstable in this genetic background. The latter may explain why it did not fix in the populations. The dynamics of the IS186 insertion in populations M3 and M4 suggest that this mutation was beneficial in the background with an IS1 insertion upstream of the homologue of *igaA*. Support for a selective advantage of this mutation is suggested by the observation that, in Salmonella, the transcription of *igaA* (*yrfF* in *E. coli*) is regulated by *lon* [37].

## Discussion

Bacterial evolution towards pathogenicity may occur through the acquisition of new genes – a gain of function mechanism- or modification of their current genomes, including loss of genes - change-of-function mechanism [38]. The later constitutes a pathoadaptation, in which mutations enhance bacterial virulence without horizontal transfer of specific genes. For example, the deletion of *hemB* in *Staphylococcus aureus* increases its ability to persist intracellularly [39] while the loss of *mucA* increases *Pseudomonas aeruginosa* ability to evade phagocytosis and resist to pulmonary clearance [40].

We followed the evolution of a commensal strain of *E. coli* under the selective pressure imposed by MΦ phagocytosis, to determine the rate of adaptive evolution and to uncover the nature of possible *E. coli* pathoadaptive mutations. From the infection dynamics and the fitness assays (Fig. 1B and 2), we conclude that at least two different adaptations, detected by the emergence different colony morphologies, occurred, namely, i) an intracellular advantage evolved by SCV clones early in the process; ii) an extracellular advantage evolved by MUC clones emerging later. The intracellular adaptation is characterized by increased bacterial resistance, plasticity and survival in the early phase of interaction with MΦ, and was accompanied by a reduced extracellular growth. The extracellular adaptation is associated with overproduction of colanic acid and characterized by increased resistance to MΦ phagocytosis. The functional link between overproduction of colanic acid and escape from phagocytosis is likely but remains to be formally established. Overtime this phenotype dominated all populations.

The mutations acquired by commensal *E. coli* adapting to MΦ, occurred within a few hundred generations and were characterized by traits reminiscent of those found in pathogenic bacteria. Clinical isolates sampled from patients suffering from recurrent and persistent infections in the blood [41] or urinary tract [21], [42], are SCVs. The distinctive traits of this phenotype are: i) ability to form small colonies, to revert to larger colony forming bacteria at high frequencies and ii) increased resistance to certain antibiotics. In *S. aureus* SCVs have been implicated as an intermediate form before mutations in *gyrA* occur to produce ciprofloxacin resistance [43]. In addition, Besier *et al.* have reported *thyA* mutant *S. aureus* SCVs show hypermutator status [44]. These findings suggest that SCVs could potentiate the emergence of mucoid clones, which latter go on to dominate the populations. However, we did not detect in SCVs the mutations found in the mucoid clones, indicating a distinct molecular basis for the SCV phenotype, an issue that we will investigate in future work. Given that mucoidy is also frequently observed in certain infections [22], [23], our finding that this trait can rapidly emerge under the selective pressure of MΦ, may have implications not only for the understanding of host-microbe interactions but also for the treatment of bacterial infections. Interestingly mucoidy can also be selected by the pressure imposed by phages in different bacterial species [45], [46]. Whether mucoid strains evolved to resist to phages also exhibit increased virulence remains to be established.

Translocation of commensal *E. coli* from the gut can be associated with severe health complications (e.g. sepsis), particularly in immunosuppressed hosts or after surgery [47], [48]. Bacteria that reach the mesenteric lymph nodes or the peritoneal cavity (extensively populated by MΦ) and that are able to escape MΦ should have a fitness advantage and potentially cause more severe disease. Indeed, we found that increased ability to escape MΦ of *in vitro* evolved clones lead to increased pathogenesis *in vivo*, when tested in a mouse model. We also found that this pathoadaptative process was characterized by three main paths. Although distinct in the number and type of mutations, these share an initial mutation: an IS insertion upstream of *yrfF*, a gene which shares 84% sequence similarity at the protein level to IgaA of *Salmonella enterica* serovar *Typhimurium*. In *S. Typhimurium* it was shown that the stability and responsiveness of the RcsCDB system depends on IgaA [49]. The RcsCDB system controls the production of colanic acid, virulence in diverse pathogens [24], [50], [51], [52], [53], modulates responses to environmental changes and is activated upon exposure to antimicrobial peptides [54], [55], [56], [57]. IgaA represses the RcsCDB system [58] and mutations causing instability of IgaA activate the RcsCDB system, leading to overproduction of colanic acid capsule (mucoid phenotype) [58]. Given the repressive function of IgaA on RcsCDB, which controls many traits likely to be important for bacterial fitness, it is likely that the observed IS insertion upstream of *yrfF* is an adaptive mutation with pleiotropic effects. If so the adaptive path may proceed through the occurrence of new mutations, which may compensate for the pleiotropic effects of that first adaptive step. Interestingly, the same amino-acid substitution in *fusA* occurred in two independent lines. *FusA* is an elongation factor and is part of the *str* operon of *E. coli*, which has 3 other genes: *rpsL*, *rpsG* and *tufA*. Since the strain that we studied carries a mutation in *rpsL* that confers streptomycin resistance, which is costly in RPMI yet increases survival inside MΦ [59], it is possible that the SNP in *fusA* could be compensatory to cost of the *rpsL* mutation in the milleu outside MΦ.

One of the adaptive paths taken by *E. coli* included insertions into the coding regions of *yiaW* and *potA* or *potD*. While the function of *yiaW* is unknown, the later genes are involved in spermidine transport, which may affect *E. coli* interaction with MΦ. Spermidines are polyamines, polycationic molecules, which interact with nucleic acids and have been described as important in escape from phagolysosomes, biofilm formation and protection from oxidative and acidic stress amongst other traits important in bacterial pathogenesis [60].

The adaptive process was also marked by the occurrence of an IS186 insertion into the promoter region of the Lon protease. Such insertion was not only likely adaptive (it was observed in two independent lineages and it increases mucoidy), but also likely leads to increased rates of transposition. Given that many of the adaptive mutations observed under the stresses imposed by MΦ were caused by ISs, these may constitute an example of Barbara McClintock proposal that transposable element movement under stress could aid organisms to adapt to new environments [61].

The mechanisms via which different mutations underlying *E. coli* pathoadaptation increase its virulence remain to be established. It is likely however, that such mechanisms would interfere with one or two host defense strategies against infections [62]. Presumably, by escaping MΦ killing pathoadaptation should provide MUC clones with a proliferative advantage, ultimately compromising host survival. This should be revealed by increased bacterial burden in the MUC infected hosts, as compared to hosts infected with non-evolved *E. coli* clones, revealing a compromise in host resistance [62]. An alternative, but not mutually exclusive, interpretation would be that pathoadaptation is associated with the induction of a immunopathologic response compromising host survival, irrespectively of pathogen burden. This should be revealed by similar bacterial burdens in the MUC infected host, as compared to hosts infected with non-evolved *E. coli* clones, revealing a compromise in host disease tolerance [62]. While critical to further understanding of the mechanism via which *E. coli* pathoadaptation increases its virulence, this is beyond the scope of the current study.

In conclusion, we demonstrate that *E. coli* can adapt to better resist to MΦ within a few hundreds of generations and that clones with different morphologies and traits similar to those of pathogenic bacteria rapidly emerge. This pathoadaptive process and the complex dynamics of the evolved phenotypes can be reasonably described by a model of clonal interference, where distinct haplotypes, carrying new transposon insertions and other mutations, increase in frequency and compete for fixation.

## Materials and Methods

### Ethics statement

All experiments involving animals were approved by the Institutional Ethics Committee at the Instituto Gulbenkian de Ciência (project nr. A009/2010 with approval date 2010/10/15), following the Portuguese legislation (PORT 1005/92) which complies with the European Directive 86/609/EEC of the European Council.

### Strains and media

The RAW 264.7 murine macrophage cell line was maintained in an atmosphere containing 5% CO_2_ at 37°C in RPMI 1640 (Gibco) supplemented with 2 mM L-glutamine (Invitrogen), 1 mM sodium pyruvate (Invitrogen), 10 mM hepes (Invitrogen), 100 U/ml penicillin/streptomycin (Gibco), 50 µM 2-mercaptoethanol solution (Gibco), 50 µg/ml gentamicin (Sigma), with 10% heat-inactivated FCS (standard RPMI complete medium). Before infection assays, MΦ were cultivated for 24 h in the same medium as before except for the three antibiotics which were now replaced by 100 µg/ml streptomycin antibiotic (RPMI-Strep medium). All bacterial cultures were also done in RPMI-Strep medium, except if stated otherwise.

The *Escherichia coli* strains used were MC4100-YFP and MC4100-CFP (MC4100, galK::CFP/YFP, Amp^R^Strep^R^) which contain the yellow (*yfp*) and cyan (*cfp*) alleles of GFP integrated at the *galK* locus in MC4100 (*E. coli* Genetic Stock Center #6152) and differ only by YFP/CFP locus that is constitutively expressed [63]. MC4100-CFP strain was used for the evolution experiment and MC4100-YFP as a reference strain for the fitness assays.

### Evolution experiment

Twelve populations were founded from a single MC4100-CFP clone and were therefore genetically uniform in the beginning of the experiments. All populations evolved in RPMI, 6 populations in the presence of the MΦ (M1–M6) and the other 6 (C1–C6) in the absence of MΦ. Before each infection cycle, MΦ (0.7×10^6^ to 1.3×10^6^/ml) were centrifuged at 1200 rpm for 5 min, re-suspended in RPMI-Strep medium and activated with 2 µg/ml CpG-ODN 1826 (5′TCCATGACGTTCCTGACGTT 3′ - Sigma) for 24 h [64]. Cells were then centrifuged (1000 rpm for 5 min), re-suspended in 3 ml of fresh RPMI-Strep medium and seeded in 12-well microtiter plates (0.8×10^6^ to 1.6×10^6^/ml). Subsequently, they were incubated at 37°C for 2 h, washed in RPMI-Strep and infected with a MOI of 1∶1 (10^6^ bacteria to 10^6^ MΦ). After 24 hours of infection, MΦ were detached with cell scraper and the whole culture was centrifuged at 4000 rpm for 10 min to pellet cells. This procedure lyses MΦ releasing intracellular bacteria. Then these were washed twice with phosphate-buffered saline (PBS) and counted by flow cytometry using a FACscan cytometer (Becton Dickinson). Approximately 10^6^ of recovered bacteria were used to infect new activated MΦ in the same manner as before. The same procedure was applied to control populations, except that 10^4^ bacteria were transferred daily. This is because after 4 hours of infection with the MΦ bacteria numbers drop to 10^4^. This adjustment results in similar number of generations in both environments. In both treatments (with and without MΦ), bacteria were allowed to propagate for approximately 15 generations per day. Generation time is estimated as: G = log_2_(Nf/Ni), where Ni is the initial number of bacteria and Nf is the final number of bacteria. Nf was approximately 6×10^8^ in both treatments. Evolution occurred during approximately 450 generations in both environments. We note that in the context of a real infection repeated contact with macrophages will not likely occur with a similar period as the one in this experimental setup.

### Fitness measurements

To estimate competitive fitness of M1–M6 populations, after 285 and 450 generations of evolution, each evolved population was competed against MC4100-YFP reference strain in the same conditions as used in the evolution experiment. Both evolved and ancestral strains were grown separately in RPMI-Strep, 10^6^ cells of each type were used to inoculate the competition plate. The initial and final ratios of both strains were determined by Flow cytometry. The fitness of each population was measured 3 times and the fitness of the ancestral strain 10 times to confirm the neutrality of the marker. A measure of relative fitness increase, expressed as selection coefficient, was estimated as:
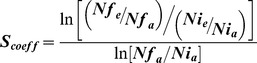

[65] where *S_coeff_* is the selective advantage of the evolved strain *e* over the ancestral strain *a*, *Nf_e_* and *Nf_a_* are the numbers of evolved (*e*) and ancestral (*a*) bacteria after competition and *Ni_a_* and *Ni_e_* are the initial numbers, before the competition.

### Dynamics of infection at 3 h post infection

Bacterial uptake was measured by the gentamicin protection assay as previously described [66], with modifications, as follows. MΦ were infected at MOI 1∶1 as described above to determine the number of intracellular and extracellular bacteria after 3 h of incubation. The number of extracellular bacteria at 3 h of incubation was estimated by taking a sample of the culture medium (without detaching the MΦ), centrifuging (4000 rpm for 10 min) to pellet the cells and finally washing these in PBS prior to plating on LB agar plates. The number of intracellular bacteria was estimated by washing infected MΦ twice with PBS and adding fresh medium containing 100 µg of gentamicin/ml to kill extracellular bacteria. After incubation for an additional hour, the medium was removed, the monolayer of macrophages was washed 3 times with PBS, detached using a cell scraper and centrifuged (4000 rpm for 10 min) to pellet the cells. These were further resuspended in PBS and the appropriate dilution was plated on LB agar plates to determine the number of intracellular bacteria. Relative abundance (Rr) of evolved clones to that of the ancestral in intracellular or extracellular environment of MΦ was estimated as:
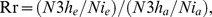
where *N3h_e_* and *N3h_a_* are the numbers of evolved (*e*) and ancestral (*a*) bacteria at 3 hours post infection (in the intracellular or extracellular niche of MΦ) and *Ni_a_* and *Ni_e_* are the initial numbers of evolved (*e*) and ancestral (*a*) bacteria used for inoculation.

To measure numbers of MΦ that are alive, the same infection protocol was performed. However, after 3 h of infection, MΦ were washed from extracellular bacteria twice with RPMI, detached and counted by Trypan blue exclusion test [67] (see Fig. S3).

### Colanic acid purification and quantification

The method used to extract colanic acid was based on a procedure described previously [68]. Briefly, 50 ml of a bacterial cell culture was heated for 15 min at 100°C to denature EPS-degrading enzymes, cooled down and centrifuged at 13200 rpm at 4°C for 30 min. Then 40 ml of the supernatant was precipitated by addition of three volumes of ethanol. The mixture was maintained at 4°C overnight and centrifuged again at 13200 rpm at 4°C for 30 min. The resulting pellet was dissolved in 5 ml of distilled water, dialyzed for 48 h against distilled water (membrane MWCO, 3500 Da) and dried in SpeedVac. Residual polypeptides were removed by precipitation with 5 ml of 10% (v/v) trichloroacetic acid and centrifuged at 13200 rpm at 4°C for 30 min. The supernatant was dialyzed for five days against distilled water and dried. The resulting preparation was resuspended in 1 ml of distilled water. Quantification of colanic acid was carried out by measuring non-dialyzable methylpentose (6-deoxy-hexose), namely fucose, which is a specific component of this exopolysaccharide. 10 to 100 µl of the colanic acid preparation were diluted to 1 ml with distilled water, and mixed with 4.5 ml of H_2_SO_4_/H_2_O (6∶1; v/v). The mixture was prepared at room temperature, then heated at 100°C for 20 min, and finally cooled down to room temperature. For each sample, absorbance at 396 nm and 427 nm was measured either directly (control sample (*A*_-co_)) or after addition of 100 µl of 0.3% freshly prepared cysteine hydrochloride (cysteine sample (*A*_-cy_)). The absorption due to the unspecific reaction with H_2_SO_4_ was subtracted from the total absorption of the sample: *A*_396-co_ and *A*_427-co_ were subtracted from A_396-cy_ and A_427-cy,_ respectively, to obtain ΔA_396_ and ΔA_427_. Values of (Δ*A*_396_–Δ*A*_427_) were directly correlated to methylpentose concentration by using a standard curve obtained with a fucose concentration ranging from 2 µg/ml to 100 µg/ml (Fig. S2).

### SCV reversion rate and auxotrophy to hemin

To determine the reversion frequency of SCV to the ancestral phenotype, single colonies grown on LB agar plates were resuspended in PBS, the appropriate dilution was plated on LB agar plates and incubated at 37°C. After 48 h small and large colonies were counted [21]. To test for the auxotrophy to hemin, individual SCV colonies were isolated, resuspended in PBS and plated on M9 minimal medium agar plates containing 2% glucose with and without hemin 50 µg/ml (Sigma-Aldrich). After incubation at 37°C for 48 h, CFUs were counted to estimate the ratio between the number of cells able to grow in presence and in absence of hemin.

### Whole genome re-sequencing and mutation prediction

Both the ancestral and 7 isolated MUC clones (MUC3_d19 sampled from population M3 after 19 days of evolution with macrophages and MUC1 to MUC6d30 sampled from M1 to M6 pops after 30 days of evolution) were grown overnight in 10 ml of RPMI-Strep at 37°C. DNA isolation from these cultures was done following a previously described protocol [69]. The DNA library construction as well as the sequencing procedure was carried out by BGI. Each sample was pair-end sequenced on an Illumina HiSeq 2000. Standard procedures produced data sets of Illumina paired-end 90 bp read pairs with insert size (including read length) of ∼470 bp.

Mutations in the two genomes were identified using the BRESEQ pipeline [70]. To detect potential duplication events we used SSAHA2 [71] and the paired-end information to map reads only to their best-match on the genome. Sequence coverage along the genome was assessed with a 250 bp window and corrected for GC% composition by normalizing by the mean coverage of regions with the same GC%. We then looked for regions with high differences (>1.4) in coverage. We did not find any such difference between the ancestral and evolved clones. See Table 2 for the identity and precise location of mutations identified in the sequenced clones. All mutations were confirmed by direct target sequencing.

### Detection of mutations

In order to determine the frequency of the mutations in clones sampled along the experiment, DNA was amplified by PCR (to identify IS insertions) and sequencing PCR was performed (to identify SNPs). DNA was amplified by PCR in a total volume of 50 µl containing 1 µl bacterial culture, 10 µM of each primer, 200 µM dNTPs, 0.5 U *Taq* polymerase and 1× *Taq* polymerase buffer. The amplification profile was 15 min at 95°C, followed by 35 cycles at 94°C for 30 s, 60°C for 90 s, 72°C for 2 min with a final extension at 72°C for 10 min. All gene fragments were amplified using these conditions and oligonucleotide primers (Table S2). The same primers were used for sequencing straight from the PCR product.

### *In-vivo* virulence tests of ancestral and evolved bacteria

We maintained male C57/BL6 mice, aged 8–10 weeks (in house supplier, Instituto Gulbenkian de Ciência), on *ad libitum* food (RM3A(P); Special Diet Services, UK) and water, with a 12 hour light cycle, at 21°C. We initiated infections by intra-peritoneal inoculation of bacteria in 100 µl saline. Several groups of mice were injected with different bacterial strains at doses ranging from 2×10^5^ to 3×10^8^ (sample sizes: ancestral – n = 46; control – n = 41, mucoid – n = 50). At doses 1×10^7^, 5×10^7^ and 1×10^8^, we injected a minimum of 10 mice, in at least two independent experiments (data from the same animals was used for the Kaplan-Meier curves in Fig. 3C). The inocula consisted of the following: a single clone for ancestral bacteria (ANC), consisted of a mixture of equal numbers of the 6 sequenced clones from day 30 (MUC1-MUC6; see Fig. 4) for the mucoids (MUC) and mixture of 6 independent clones evolved in the absence of macrophages (CON). Furthermore, as a control, in each experimental block we injected a group of 2–3 mice with 100 µl of saline (these animals did not display any signs of disease). We monitored mice for a period of 6–10 days (twice a day for the first two days and daily for the remaining 8 days) and measured weight and temperature.

To estimate the LD_50_ values (Fig. 3A–B,E), we fitted a binomial generalized linear model (GLM) for each morphotype, using survival as a response variable and log_10_ bacterial dose as explanatory variable (following [72]). To analyze the temporal dynamics of mortality in mice infected with MUC or ANC at the MUC LD_50_ (Fig. 3C), we used Kaplan-Meier curves followed by a log-rank test. Finally, we used GLMs to test whether the variation maximum reduction in temperature or weight could be explained by the infecting strain.

### Statistical analysis

The statistical analysis was performed using the R software: http://www.r-project.org/.

## Supporting Information

Figure S1
**Infection dynamics of the ancestral strain.** Variation in numbers of bacteria (A) and MΦ (B) during an infection with the ancestral clone (ANC) at MOI (1∶1). (C) Simulated dynamics of a population of ancestral bacteria dividing in the presence of MΦ for 24 hours, following the deterministic model 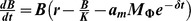 (see Text S1), with the following parameter values: *B*_0_ = 10^6^; *M* **_Φ_** = 10^6^; *r* = 2.3; *K* = 10^8^; *a_m_* = −3.7*10^−6^; δ = 0.1. We assume that MΦ decay at linear rate of 0.1 following the data of (B).(TIF)

Figure S2 **Calibration curve for quantification of colanic acid in evolved clones.** Empty circles represent three replicate measurements and filled circles their average.
(TIF)

Figure S3 **Comparison of MUC and ANC clones in the presence of MΦ.** (A) Numbers of alive MΦ after 3 h of infection with evolved MUC clones versus ANC clone. (B) MUC clones survive inside MΦ similarly to ANC clone: in the Y-axis it is shown the percentage of alive bacteria after 24 hours of internalization. (C) MUC clones do not impair MΦ ability to engulf ANC bacteria. Co-infection was performed with ANC and MUC clones (a pool of six sequenced MUC1 to MUC6 clones at equal frequencies). Black bars represent bacteria used for initial inoculation (0 h) and grey bars – bacteria recovered intracellularly after 3 h of infection (3 h). Error bars correspond to 2SE.
(TIF)

Figure S4 **Levels of the pro-inflammatory cytokine TNF.** The level of TNF detected after 4 hours of MΦ infection with ANC, CON and MUC bacteria at MOI = 0.01. Means of 3 independent experiments are shown with error bars corresponding to SEM. NI- not infected macrophages.
(TIF)

Figure S5 **Growth of evolved clones in the presence of polyamines.** The ratio between carrying capacity (K) in the presence and absence of spermidine (A) or spermine (B) after 24 hours of growth indicate differential survival in the presence of polyamines. MUC1 to MUC6 correspond to MUC_M1_D30 to MUC_M6_D30 clones and MUC3* corresponds to MUC_M3_D19 clone. Error bars correspond to 2SE.
(TIF)

Figure S6 **Estimation of mutation frequency of ancestral strain (ANC) in the presence and in the absence of MΦ.** There was no difference in the frequency of nitrofuran-resistant clones in the presence versus absence of MΦ (median frequency 5.25×10^−6^ vs. 4.35×10^−6^, P = 0.49, W = 78, Mann-Whitney U test), as well as there was no difference in the insertion frequency (with MΦ IS frequency was 52% (51 out of 98 sampled clones), without MΦ IS frequency was 59% (50 out of 85 sampled clones)).
(TIF)

Figure S7 **Variation in exopolysaccharide production among evolved clones.** Amount of EPS per bacterial cell was measured for the ancestral strain (ANC) and six mucoid clones that evolved independently (MUC1 to MUC6): we also measured the amount of EPS in MUC_M3_D19 (MUC3*) and in six other clones derived from this clone after a growth in RPMI (T136–T138, T122–T124). None of these derived clones have the IS186 insertion in *lon* promoter region and all have the IS1 insertion upstream of *yrfF*. T136–T138 are visibly mucoid and T122–T124 show a non-mucoid colony morphology. All measurements were done in triplicate.
(TIF)

Figure S8 **Relative abundance of evolved clones in the presence of MΦ.** Relative abundance (R_r_) of MUC_M3_D19 (MUC) clone and six clones derived from this clone (T122–T124 non-mucoid and T136–T138 mucoid clones) are represented either inside MΦ (black bars) or outside MΦ (white bars).
(TIFF)

Figure S9 **Dynamics for the different haplotypes under the model of clonal interference.** Simulated frequencies of the different haplotypes which result in the frequencies of the mucoid phenotypes of Figure 5. *r* = 2.3, am  = −3.7×10^−6^ and the other parameters used are shown in Table S3. In this table, the cases where more haplotypes were assumed to reproduce the experimental dynamics are marked with *, and the additional parameters are in Table S4.
(TIFF)

Figure S10
**Region of parameter space theoretically expected for the invasion of first mucoid morph.** Colored areas show the parameter region (*r_m_/r* and *a_mmuc_*/*a_m_*) where a mucoid genotype (mimicking the IS insertion upstream of *yrfF* in the experiment) that has emerged is able to increase in frequency so that it can survive the bottleneck imposed every 24 hours in the experiment. The equations for these simulations are:
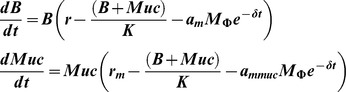
with initial conditions Muc(0) = 1, B(0) = 10^6^ and the other parameter values as in Figure S1: *M*_Φ_ = 10^6^; *r* = 2.3; *K* = 10^8^; *a_m_* = 3.7*10^−6^; δ = 0.1. Note that the escape parameter is negative (according to the mathematical model) and, therefore, a value lower than 1 indicates a higher ability to escape predation. Warmer colors show higher frequency of the mucoid genotype in the population after 24 hours of its emergence as a single copy. The black dot indicates the value of *r_m_* and *a_mmuc_*, of the first mucoid haplotype assumed to emerge in the 6 models that produced the dynamics in Figures 5 and S8.(TIFF)

Figure S11 **Relative growth rate for clones with an ancestral colony morphology (non-mucoid).** Multiple clones were randomly isolated from two populations at different time points, as indicated in the x-axis, below the clone numbers. Replicate measures for the maximum growth rate of each clone were obtained from independent cultures and divided by the mean growth rate of the original ancestral. The ancestral for the main experiment (ANC) is highlighted in red, the evolved clones whose growth rate is significantly different from the ancestral are highlighted in blue (P<0.05, ANOVA; white: not significantly different from ANC). ANC: 16 replicates; evolved clones: 3–4 replicates.
(TIFF)

Figure S12 **Fluctuation test of evolved MUC clone (MUC_M3_D19) and ANC clone to test for increase in mutagenesis.** Black squares (for MUC clone) and white squares (for ANC clone) each represent 50 independent measurements of the frequency of spontaneous mutants resistant to d-cycloserine. Mutation rates were significantly higher in the MUC compared with the ANC clone (median MUC = 2.6×10^−6^ and ANC = 1×10^−7^, P = 5.5×10^−13^, W = 203.5, Mann-Whitney U test).
(TIFF)

Protocol S1 **Full script for the model of clonal interference obtained in Mathematica v8.0.**
(PDF)

Table S1 **Increased resistance of the SCV clone to aminoglycoside antibiotics.** Minimal inhibitory concentration (MIC) of each clone was measured in triplicate by a disc diffusion assay. S indicates sensitive clones and R resistant clones. SCV_M1_D8, SCV_M2_D4, SCV_M3_D5 and MUC_M2_D19, MUC_M3_D19, MUC_M4_D19 clones are shown.
(DOC)

Table S2 **Primers used in this study.**
(DOC)

Table S3 **Parameters used in modeling the dynamics of the different haplotypes.** Parameters used for the dynamics in figure S9. Cases where more haplotypes were assumed to reproduce the experimental dynamics are marked with *, and the additional parameters are in Table S4.
(DOC)

Table S4 **Parameters for the additional haplotypes for the modeled dynamics.** Parameters in the additional haplotypes required to obtain the dynamics in Figure S9.
(DOC)

Text S1 **Includes Model of Clonal interference, Supplementary Materials and Methods and Supplementary References.**
(DOC)
