# Supplementary figures and images for: The Genetic Basis of Escherichia coli Pathoadaptation to Macrophages
Source: PLoS Pathog. 2013 Dec 12;9(12):e1003802. doi: 10.1371/journal.ppat.1003802 (PMC3861542; doi:10.1371/journal.ppat.1003802)

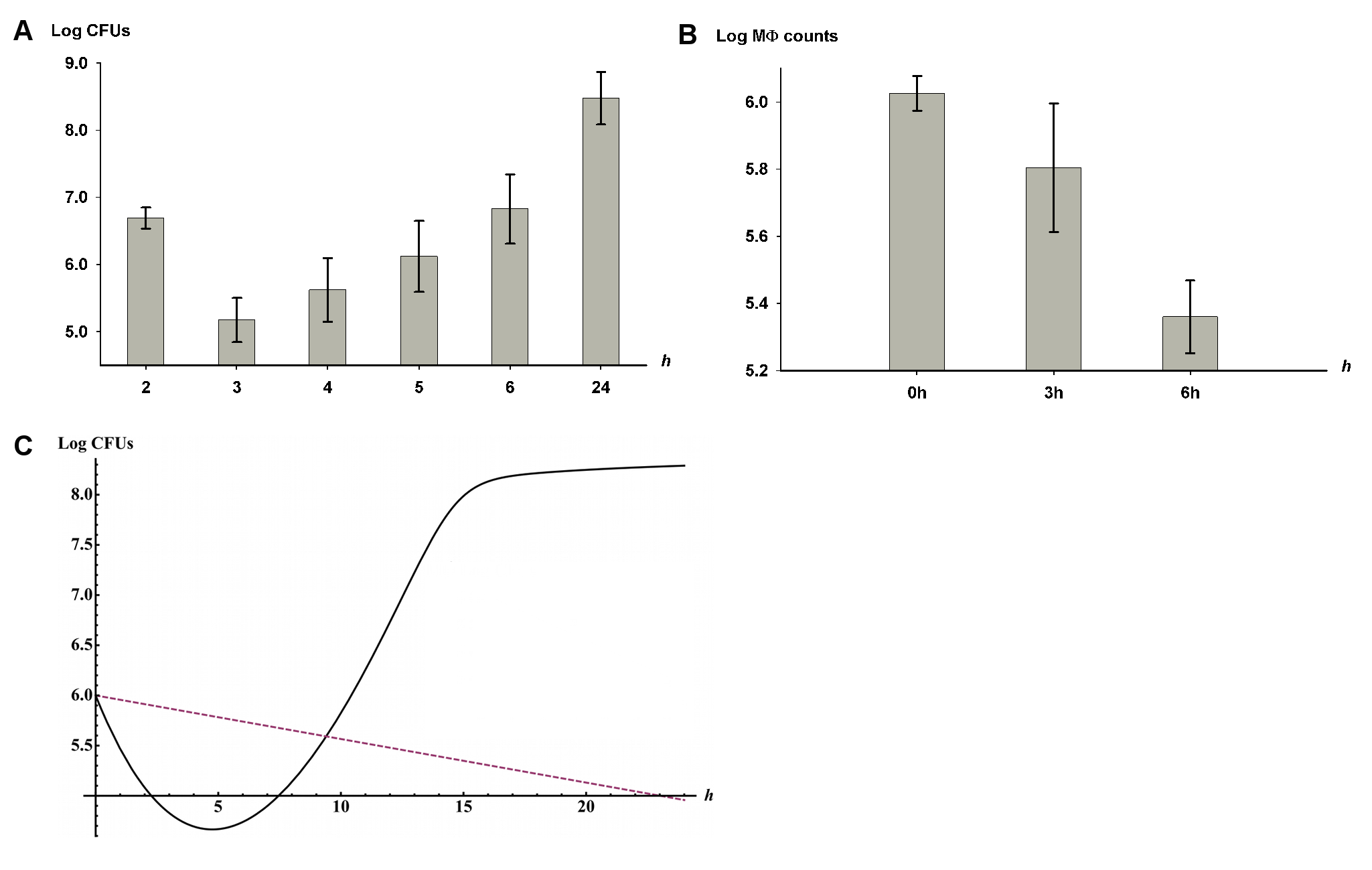

Supplement: Figure S1 — Infection dynamics of the ancestral strain. Variation in numbers of bacteria (A) and MΦ (B) during an infection with the ancestral clone (ANC) at MOI (1∶1). (C) Simulated dynamics of a population of ancestral bacteria dividing in the presence of MΦ for 24 hours, following the deterministic model (see Text S1), with the following parameter values: B 0 = 106; M Φ = 106; r = 2.3; K = 108; am = −3.7*10−6; δ = 0.1. We assume that MΦ decay at linear rate of 0.1 following the data of (B). (TIF) [file ppat.1003802.s001.tif]

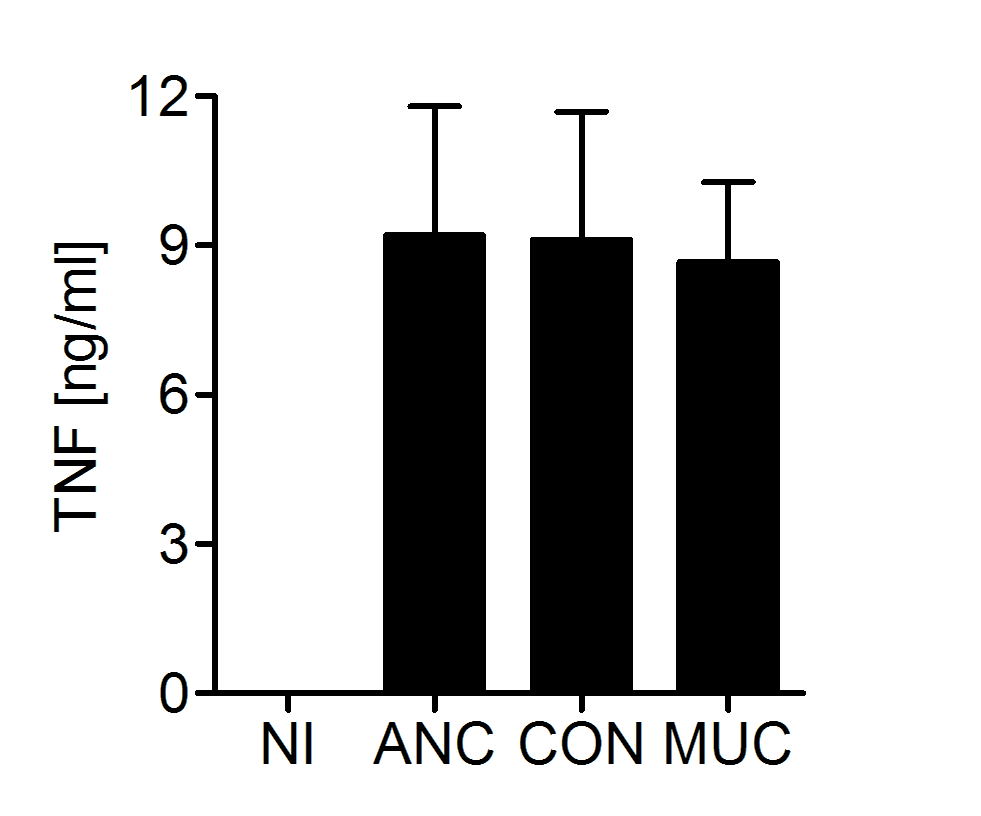

Supplement: Figure S4 — Levels of the pro-inflammatory cytokine TNF. The level of TNF detected after 4 hours of MΦ infection with ANC, CON and MUC bacteria at MOI = 0.01. Means of 3 independent experiments are shown with error bars corresponding to SEM. NI- not infected macrophages. (TIF) [file ppat.1003802.s004.tif]

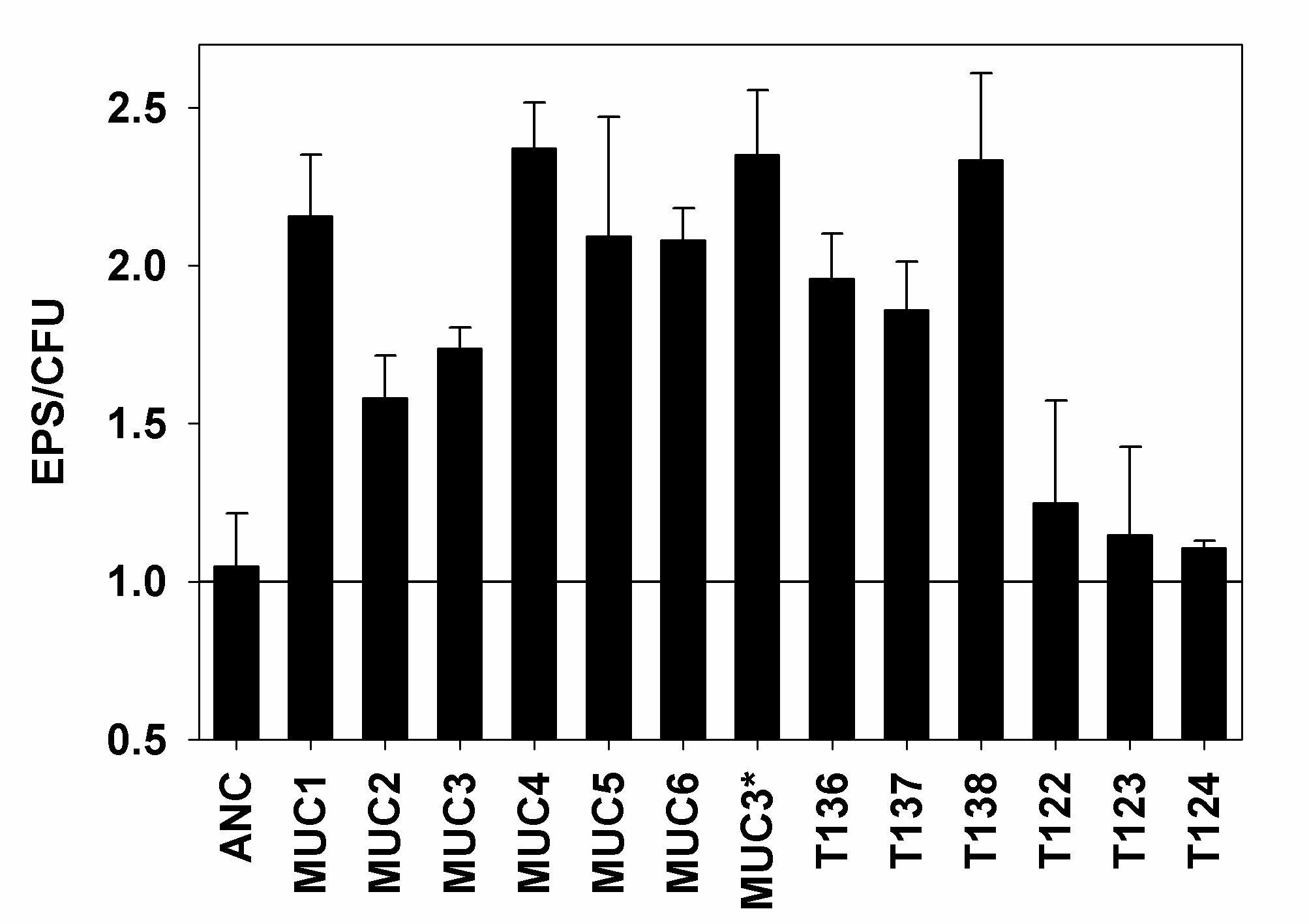

Supplement: Figure S7 — Variation in exopolysaccharide production among evolved clones. Amount of EPS per bacterial cell was measured for the ancestral strain (ANC) and six mucoid clones that evolved independently (MUC1 to MUC6): we also measured the amount of EPS in MUC_M3_D19 (MUC3*) and in six other clones derived from this clone after a growth in RPMI (T136–T138, T122–T124). None of these derived clones have the IS186 insertion in lon promoter region and all have the IS1 insertion upstream of yrfF. T136–T138 are visibly mucoid and T122–T124 show a non-mucoid colony morphology. All measurements were done in triplicate. (TIF) [file ppat.1003802.s007.tif]

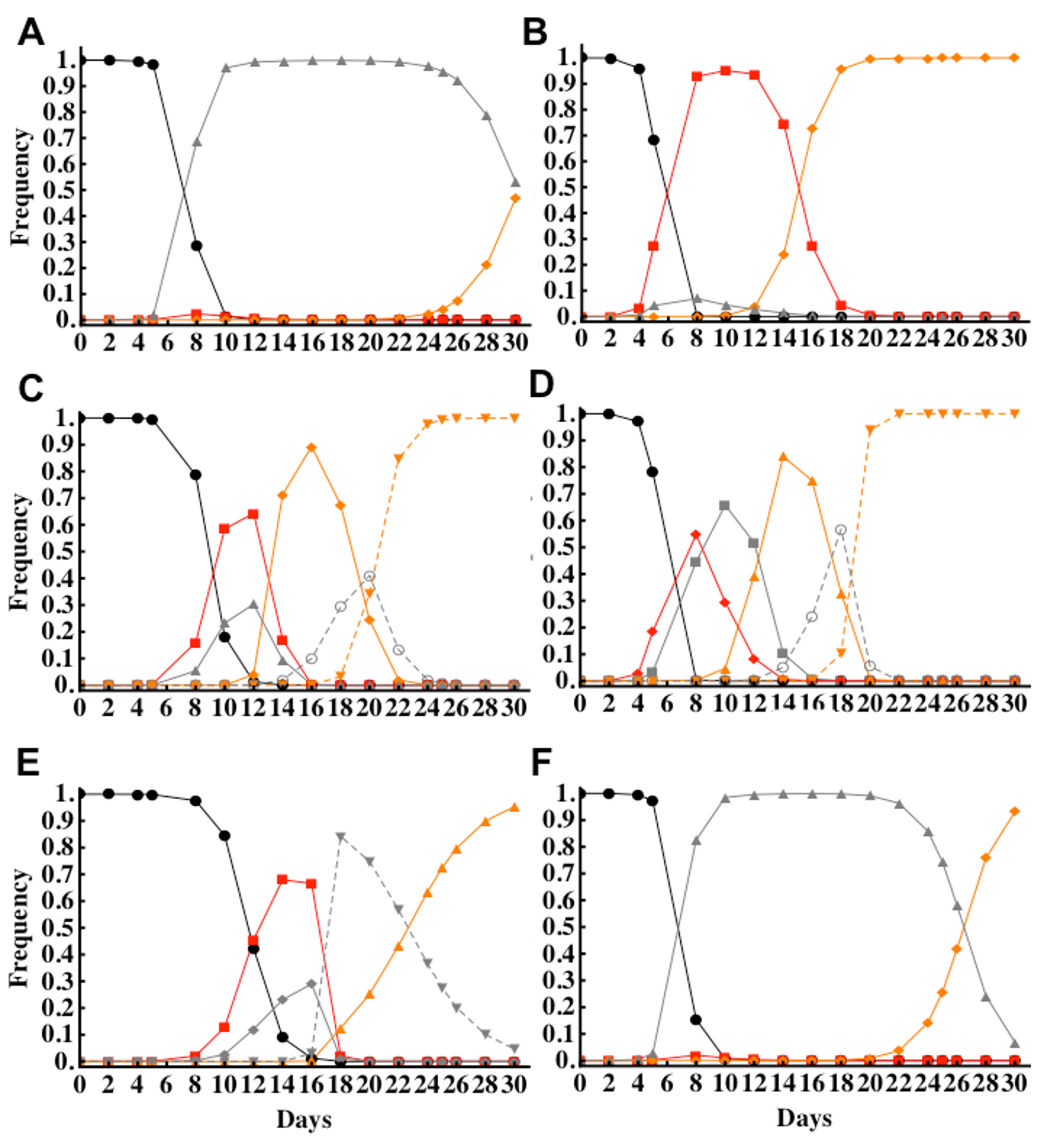

Supplement: Figure S9 — Dynamics for the different haplotypes under the model of clonal interference. Simulated frequencies of the different haplotypes which result in the frequencies of the mucoid phenotypes of Figure 5. r = 2.3, am = −3.7×10^−6^ and the other parameters used are shown in Table S3. In this table, the cases where more haplotypes were assumed to reproduce the experimental dynamics are marked with *, and the additional parameters are in Table S4. (TIFF) [file ppat.1003802.s009.tiff]

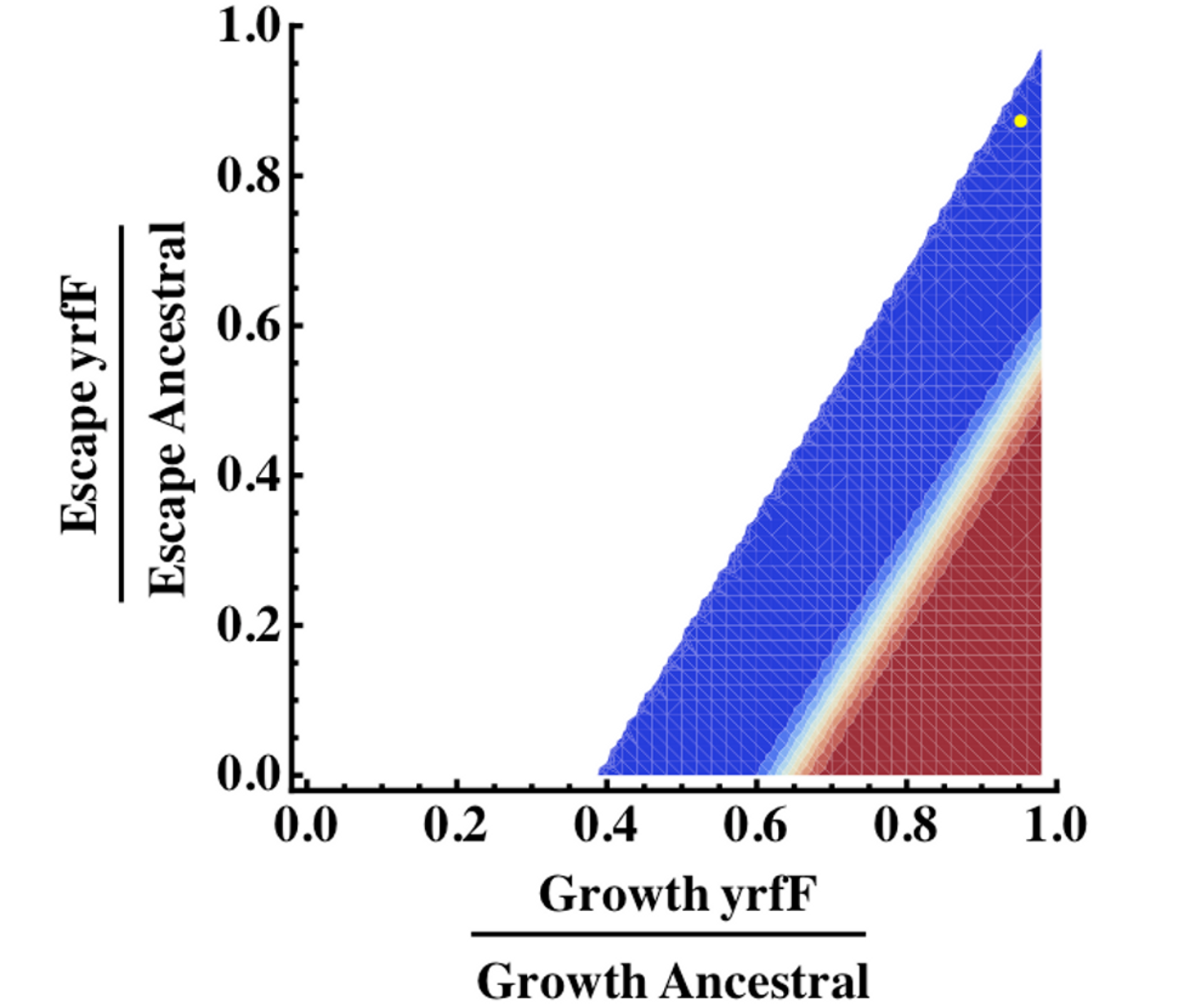

Supplement: Figure S10 — Region of parameter space theoretically expected for the invasion of first mucoid morph. Colored areas show the parameter region (rm/r and ammuc/am) where a mucoid genotype (mimicking the IS insertion upstream of yrfF in the experiment) that has emerged is able to increase in frequency so that it can survive the bottleneck imposed every 24 hours in the experiment. The equations for these simulations are:with initial conditions Muc(0) = 1, B(0) = 106 and the other parameter values as in Figure S1: M Φ = 106; r = 2.3; K = 108; am = 3.7*10−6; δ = 0.1. Note that the escape parameter is negative (according to the mathematical model) and, therefore, a value lower than 1 indicates a higher ability to escape predation. Warmer colors show higher frequency of the mucoid genotype in the population after 24 hours of its emergence as a single copy. The black dot indicates the value of rm and ammuc, of the first mucoid haplotype assumed to emerge in the 6 models that produced the dynamics in Figures 5 and S8. (TIFF) [file ppat.1003802.s010.tiff]

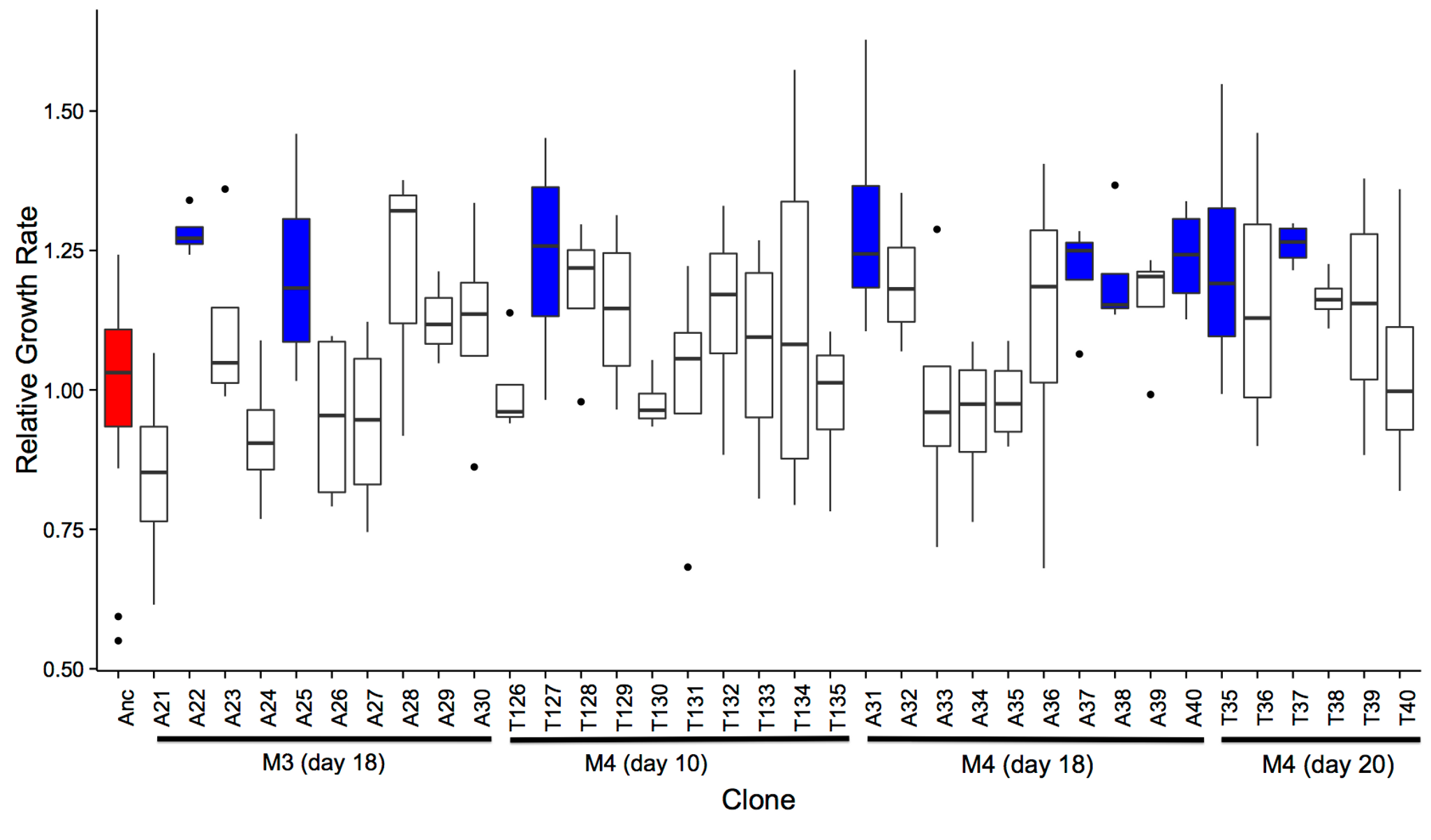

Supplement: Figure S11 — Relative growth rate for clones with an ancestral colony morphology (non-mucoid). Multiple clones were randomly isolated from two populations at different time points, as indicated in the x-axis, below the clone numbers. Replicate measures for the maximum growth rate of each clone were obtained from independent cultures and divided by the mean growth rate of the original ancestral. The ancestral for the main experiment (ANC) is highlighted in red, the evolved clones whose growth rate is significantly different from the ancestral are highlighted in blue (P<0.05, ANOVA; white: not significantly different from ANC). ANC: 16 replicates; evolved clones: 3–4 replicates. (TIFF) [file ppat.1003802.s011.tiff]
