# Supplementary material for: The Genetic Basis of Escherichia coli Pathoadaptation to Macrophages
Source: PLoS Pathog. 2013 Dec 12;9(12):e1003802. doi: 10.1371/journal.ppat.1003802 (PMC3861542; doi:10.1371/journal.ppat.1003802)
Supplement: Protocol S1 — Full script for the model of clonal interference obtained in Mathematica v8.0. (PDF) [file ppat.1003802.s013.pdf]

```
ClearAll["Global`*"]

PlotFunction[ExperData_, SimData_] := Table[{

days = {0, 2, 4, 5, 8, 10, 12, 14, 16, 18, 20, 22, 24, 25, 26, 28, 30};

ListLinePlot[{Table[{days[[i]], ExperData[[1]][[i]]}, {i, 1, 17}],
      Table[{days[[i]], ExperData[[3]][[i]]}, {i, 1, 17}]},
     Mesh -> Full, PlotStyle -> {Dashed, Black}, PlotMarkers -> Automatic,
     PlotRange -> {{0, 31}, {-0.02, 1}}, Frame -> {{True, False}, {True, False}},
     FrameLabel -> {"Days", "Frequency"},
     FrameTicks -> {Table[i, {i, 0, 30, 2}], Table[i, {i, 0, 1, 0.1}]}],

 ListLinePlot[{Table[{days[[i]], SimData[[2]][[i]]}, {i, 1, 17}]}, Mesh -> Full,
     PlotStyle -> {{Black, Dashed}, Black, {Red, Dashed}, Red},
     PlotMarkers -> {Automatic, Medium}, PlotRange -> {{0, 31}, {-0.02, 1.05}},
     Frame -> {{True, False}, {True, False}},
     FrameLabel -> {"Days", "Frequency"},
     FrameTicks -> {Table[i, {i, 0, 30, 2}], Table[i, {i, 0, 1, 0.1}]},
     FrameStyle -> {{Thick, Thick}, {Thick, Thick}},
     BaseStyle -> {Bold, FontSize -> 18}],

ListLinePlot[{Table[{days[[i]], SimData[[3]][[i]]}, {i, 1, 17}],
      Table[{days[[i]], SimData[[4]][[i]]}, {i, 1, 17}],
      Table[{days[[i]], SimData[[5]][[i]]}, {i, 1, 17}],
      Table[{days[[i]], SimData[[6]][[i]]}, {i, 1, 17}]},
     Mesh -> Full, PlotStyle -> {Black, Red, Orange, Gray},
     PlotMarkers -> {Automatic, Medium}, PlotRange -> {{0, 31}, {-0.02, 1.05}},
     Frame -> {{True, False}, {True, False}}, FrameLabel -> {"Days", "Frequency"},
     FrameTicks -> {Table[i, {i, 0, 30, 2}], Table[i, {i, 0, 1, 0.1}]},
     FrameStyle -> {{Thick, Thick}, {Thick, Thick}},
     BaseStyle -> {Bold, FontSize -> 18}]}];

EvolveFunctionM1[U_?NumericQ, Ute_?NumericQ, factorGrowthImpAnc_?NumericQ,
  factorGrowthMuc_?NumericQ, factorGrowthImpMuc_?NumericQ,
factorPredationMuc_?NumericQ] :=

Module[{results = {}, mucoidOverDays = {},
   nonmucoidOverDays = {}, ancestralOverDays = {},
   impAncOverDays = {}, mucOverDays = {}, impMucOverDays = {},
i, t, assayedDays,
Daytime = 24, Exptime = 31, limiting = 10^-8, bGrowthBase = 2.3,
   predationBase = -3.7 * 10^-6, m0 = 10^6, mGrowth = 0.1,
   ancestral = 10^6, mutantMuc = 0, mutantImpAnc = 0, mutantImpMuc = 0,
ance, mutaMuc, mutaImpAnc, mutaImpMuc, growthMuc, growthImpAnc,
   growthImpMuc, predationMuc, predationImpAnc,
   predationImpMuc, freqAnc, freqMuc, freqImpAnc, freqImpMuc},

(*Mucoid derived from ancestral*)
growthMuc = bGrowthBase * factorGrowthMuc;
  (*COST Factor should be between 0.9 and 1*)
predationMuc = predationBase * factorPredationMuc;
  (*ADVANTAGE Factor should be between 0.8 and 1*)

(*Improved ancestral*)
```

```
growthImpAnc = bGrowthBase * factorGrowthImpAnc;
  (*ADVANTAGE Factor should be between 1 and 1.1*)
predationImpAnc = predationBase; (*FIXED Should not change from ancestral*)

(*Improved Mucoid*)
growthImpMuc = growthMuc * factorGrowthImpMuc;
  (*ADVANTAGE Factor should be between 1 and 1.1*)
predationImpMuc = predationMuc;
  (*FIXED Should not change from first mucoid*)

assayedDays = {0, 2, 4, 5, 8, 10, 12, 14, 16, 18, 20, 22, 24, 25, 26, 28, 30};

For [i = 1, i < Exptime, i++,
s = NDSolve[
{
ance'[t] == ance[t] * (bGrowthBase -
            limiting * (ance[t] + mutaMuc[t] + mutaImpAnc[t] + mutaImpMuc[t]) +
            predationBase * m0 * Exp[-mGrowth * t]) - (U - Ute) * ance[t],

mutaMuc'[t] == mutaMuc[t] * (growthMuc - limiting *
             (ance[t] + mutaMuc[t] + mutaImpAnc[t] + mutaImpMuc[t]) + predationMuc *
             m0 * Exp[-mGrowth * t]) + Ute * ance[t] - 4 * U * mutaMuc[t],

mutaImpAnc'[t] == mutaImpAnc[t] * (growthImpAnc -
            limiting * (ance[t] + mutaMuc[t] + mutaImpAnc[t] + mutaImpMuc[t]) +
            predationImpAnc * m0 * Exp[-mGrowth * t]) + U * ance[t],

mutaImpMuc'[t] == mutaImpMuc[t] * (growthImpMuc -
            limiting * (ance[t] + mutaMuc[t] + mutaImpAnc[t] + mutaImpMuc[t]) +
            predationImpMuc * m0 * Exp[-mGrowth * t]) + 4 * U * mutaMuc[t],

ance[0] == ancestral, mutaMuc[0] == mutantMuc,
      mutaImpAnc[0] == mutantImpAnc, mutaImpMuc[0] == mutantImpMuc}

, {ance, mutaMuc, mutaImpAnc, mutaImpMuc}
, {t, Daytime}];

ancestral = ance[Daytime] /. s; mutantMuc = mutaMuc[Daytime] /. s; mutantImpAnc =
    mutaImpAnc[Daytime] /. s; mutantImpMuc = mutaImpMuc[Daytime] /. s;

freqMuc = mutantMuc[[1]] /
     (mutantMuc[[1]] + ancestral[[1]] + mutantImpAnc[[1]] + mutantImpMuc[[1]]);
freqImpAnc = mutantImpAnc[[1]] / (mutantMuc[[1]] + ancestral[[1]] +
       mutantImpAnc[[1]] + mutantImpMuc[[1]]);
freqImpMuc = mutantImpMuc[[1]] / (mutantMuc[[1]] + ancestral[[1]] +
       mutantImpAnc[[1]] + mutantImpMuc[[1]]);
freqAnc = 1 - (freqMuc + freqImpAnc + freqImpMuc);

mutantMuc = Floor[freqMuc * 10^6]; mutantImpAnc = Floor[freqImpAnc * 10^6];
   mutantImpMuc = Floor[freqImpMuc * 10^6]; ancestral = Floor[freqAnc * 10^6];


If[i == 1,
impAncOverDays = Append[impAncOverDays, 0];
mucOverDays = Append[mucOverDays, 0];
```

```
impMucOverDays = Append[impMucOverDays, 0];
ancestralOverDays = Append[ancestralOverDays, 1];

mucoidOverDays = Append[mucoidOverDays, (freqMuc + freqImpMuc)];
nonmucoidOverDays = Append[nonmucoidOverDays, (freqAnc + freqImpAnc)];
]


If[Count[assayedDays, i] > 0,
impAncOverDays = Append[impAncOverDays, freqImpAnc];
mucOverDays = Append[mucOverDays, freqMuc];
impMucOverDays = Append[impMucOverDays, freqImpMuc];
ancestralOverDays = Append[ancestralOverDays, ancestral / 10^6];

mucoidOverDays = Append[mucoidOverDays, (freqMuc + freqImpMuc)];
nonmucoidOverDays = Append[nonmucoidOverDays, (freqAnc + freqImpAnc)];
]
];
results = Append[results, nonmucoidOverDays];
 results = Append[results, mucoidOverDays];

results = Append[results, ancestralOverDays];
results = Append[results, mucOverDays];
results = Append[results, impMucOverDays];
results = Append[results, impAncOverDays];
results]



EvolveFunctionM2[U_?NumericQ, Ute_?NumericQ, factorGrowthImpAnc_?NumericQ,
  factorGrowthMuc_?NumericQ, factorGrowthImpMuc_?NumericQ,
factorPredationMuc_?NumericQ] :=

Module[{results = {}, mucoidOverDays = {},
   nonmucoidOverDays = {}, ancestralOverDays = {},
   impAncOverDays = {}, mucOverDays = {}, impMucOverDays = {},
i, t, assayedDays,
Daytime = 24, Exptime = 31, limiting = 10^-8, bGrowthBase = 2.3,
   predationBase = -3.7 * 10^-6, m0 = 10^6, mGrowth = 0.1,
   ancestral = 10^6, mutantMuc = 0, mutantImpAnc = 0, mutantImpMuc = 0,
ance, mutaMuc, mutaImpAnc, mutaImpMuc, growthMuc, growthImpAnc,
   growthImpMuc, predationMuc, predationImpAnc,
   predationImpMuc, freqAnc, freqMuc, freqImpAnc, freqImpMuc},

(*Mucoid derived from ancestral*)
growthMuc = bGrowthBase * factorGrowthMuc;
  (*COST Factor should be between 0.9 and 1*)
predationMuc = predationBase * factorPredationMuc;
  (*ADVANTAGE Factor should be between 0.8 and 1*)

(*Improved ancestral*)
growthImpAnc = bGrowthBase * factorGrowthImpAnc;
  (*ADVANTAGE Factor should be between 1 and 1.1*)
predationImpAnc = predationBase; (*FIXED Should not change from ancestral*)
```

```
(*Improved Mucoid*)
growthImpMuc = growthMuc * factorGrowthImpMuc;
  (*ADVANTAGE Factor should be between 1 and 1.1*)
predationImpMuc = predationMuc;
  (*FIXED Should not change from first mucoid*)

assayedDays = {0, 2, 4, 5, 8, 10, 12, 14, 16, 18, 20, 22, 24, 25, 26, 28, 30};

For [i = 1, i < Exptime, i++,
s = NDSolve[
{
ance'[t] == ance[t] * (bGrowthBase -
            limiting * (ance[t] + mutaMuc[t] + mutaImpAnc[t] + mutaImpMuc[t]) +
            predationBase * m0 * Exp[-mGrowth * t]) - (U + Ute) * ance[t],

mutaMuc'[t] == mutaMuc[t] * (growthMuc - limiting *
              (ance[t] + mutaMuc[t] + mutaImpAnc[t] + mutaImpMuc[t]) + predationMuc *
              m0 * Exp[-mGrowth * t]) + Ute * ance[t] - U * 4 * mutaMuc[t],

mutaImpAnc'[t] == mutaImpAnc[t] * (growthImpAnc -
            limiting * (ance[t] + mutaMuc[t] + mutaImpAnc[t] + mutaImpMuc[t]) +
            predationImpAnc * m0 * Exp[-mGrowth * t]) + U * ance[t],

mutaImpMuc'[t] == mutaImpMuc[t] * (growthImpMuc -
            limiting * (ance[t] + mutaMuc[t] + mutaImpAnc[t] + mutaImpMuc[t]) +
            predationImpMuc * m0 * Exp[-mGrowth * t]) + U * 4 * mutaMuc[t],

ance[0] == ancestral, mutaMuc[0] == mutantMuc,
      mutaImpAnc[0] == mutantImpAnc, mutaImpMuc[0] == mutantImpMuc}

, {ance, mutaMuc, mutaImpAnc, mutaImpMuc}
, {t, Daytime}];

ancestral = ance[Daytime] /. s; mutantMuc = mutaMuc[Daytime] /. s; mutantImpAnc =
    mutaImpAnc[Daytime] /. s; mutantImpMuc = mutaImpMuc[Daytime] /. s;

freqMuc = mutantMuc[[1]] /
      (mutantMuc[[1]] + ancestral[[1]] + mutantImpAnc[[1]] + mutantImpMuc[[1]]);
freqImpAnc = mutantImpAnc[[1]] / (mutantMuc[[1]] + ancestral[[1]] +
       mutantImpAnc[[1]] + mutantImpMuc[[1]]);
freqImpMuc = mutantImpMuc[[1]] / (mutantMuc[[1]] + ancestral[[1]] +
       mutantImpAnc[[1]] + mutantImpMuc[[1]]);
freqAnc = 1 - (freqMuc + freqImpAnc + freqImpMuc);

mutantMuc = Floor[freqMuc * 10^6]; mutantImpAnc = Floor[freqImpAnc * 10^6];
   mutantImpMuc = Floor[freqImpMuc * 10^6]; ancestral = Floor[freqAnc * 10^6];


If[i == 1,
impAncOverDays = Append[impAncOverDays, 0];
mucOverDays = Append[mucOverDays, 0];
impMucOverDays = Append[impMucOverDays, 0];
ancestralOverDays = Append[ancestralOverDays, 1];

mucoidOverDays = Append[mucoidOverDays, (freqMuc + freqImpMuc)];
```

```
nonmucoidOverDays = Append[nonmucoidOverDays, (freqAnc + freqImpAnc)];
]



If[Count[assayedDays, i] > 0,
impAncOverDays = Append[impAncOverDays, freqImpAnc];
mucOverDays = Append[mucOverDays, freqMuc];
impMucOverDays = Append[impMucOverDays, freqImpMuc];
ancestralOverDays = Append[ancestralOverDays, ancestral / 10^6];

mucoidOverDays = Append[mucoidOverDays, (freqMuc + freqImpMuc)];
nonmucoidOverDays = Append[nonmucoidOverDays, (freqAnc + freqImpAnc)];
]
];
results = Append[results, nonmucoidOverDays];
 results = Append[results, mucoidOverDays];

results = Append[results, ancestralOverDays];
results = Append[results, mucOverDays];
results = Append[results, impMucOverDays];
results = Append[results, impAncOverDays];
results]


EvolveFunctionM3[U_?NumericQ, Ute_?NumericQ, factorGrowthImpAnc_?NumericQ,
  factorGrowthMuc_?NumericQ, factorGrowthImpMuc_?NumericQ,
  factorGrowthSecImpMuc_?NumericQ, factorGrowthSecImpAnc_?NumericQ,
factorPredationMuc_?NumericQ] :=

Module[{results = {}, mucoidOverDays = {}, nonmucoidOverDays = {},
   ancestralOverDays = {}, impAncOverDays = {}, mucOverDays = {},
   impMucOverDays = {}, secImpMucOverDays = {}, secImpAncOverDays = {},
i, t, assayedDays,
Daytime = 24, Exptime = 31, limiting = 10^-8,
   bGrowthBase = 2.3, predationBase = -3.7 * 10^-6, m0 = 10^6,
   mGrowth = 0.1, ancestral = 10^6, mutantMuc = 0, mutantImpAnc = 0,
   mutantImpMuc = 0, mutantSecImpMuc = 0, mutantSecImpAnc = 0,
ance, mutaMuc, mutaImpAnc, mutaImpMuc, mutaSecImpAnc, mutaSecImpMuc,
   growthMuc, growthImpAnc, growthImpMuc, growthSecImpMuc,
   growthSecImpAnc, predationMuc, predationImpAnc, predationImpMuc,
   predationSecImpMuc, predationSecImpAnc, freqAnc, freqMuc,
   freqImpAnc, freqImpMuc, freqSecImpMuc, freqSecImpAnc},

(*Mucoid derived from ancestral*)
growthMuc = bGrowthBase * factorGrowthMuc;
  (*COST Factor should be between 0.9 and 1*)
predationMuc = predationBase * factorPredationMuc;
  (*ADVANTAGE Factor should be between 0.8 and 1*)

(*Improved ancestral*)
growthImpAnc = bGrowthBase * factorGrowthImpAnc;
  (*ADVANTAGE Factor should be between 1 and 1.1*)
predationImpAnc = predationBase; (*FIXED Should not change from ancestral*)
```

```
(*Improved Mucoid*)
growthImpMuc = growthMuc * factorGrowthImpMuc;
  (*ADVANTAGE Factor should be between 1 and 1.1*)
predationImpMuc = predationMuc;
  (*FIXED Should not change from first mucoid*)

(*Second Improved Mucoid*)
growthSecImpMuc = growthImpMuc;
  (*ADVANTAGE Factor should be between 1 and 1.1*)
predationSecImpMuc = predationMuc * factorGrowthSecImpMuc;
  (*FIXED Should not change from first mucoid*)

(*Second Improved Ancestral*)
growthSecImpAnc = growthImpAnc * factorGrowthSecImpAnc;
  (*ADVANTAGE Factor should be between 1 and 1.1*)
predationSecImpAnc = predationBase;
  (*FIXED Should not change from first mucoid*)


assayedDays = {0, 2, 4, 5, 8, 10, 12, 14, 16, 18, 20, 22, 24, 25, 26, 28, 30};

For [i = 1, i < Exptime, i++,
s = NDSolve[
{
ance'[t] ==
        ance[t] * (bGrowthBase - limiting * (ance[t] + mutaMuc[t] + mutaImpAnc[t] +
                mutaImpMuc[t] + mutaSecImpAnc[t] + mutaSecImpMuc[t]) +
             predationBase * m0 * Exp[-mGrowth * t]) - (U + Ute) * ance[t],

mutaImpAnc'[t] == mutaImpAnc[t] * (growthImpAnc -
             limiting * (ance[t] + mutaMuc[t] + mutaImpAnc[t] + mutaImpMuc[t] +
                mutaSecImpAnc[t] + mutaSecImpMuc[t]) + predationImpAnc *
              m0 * Exp[-mGrowth * t]) + U * ance[t] - U * mutaImpAnc[t],

mutaSecImpAnc'[t] == mutaSecImpAnc[t] *
           (growthSecImpAnc - limiting * (ance[t] + mutaMuc[t] + mutaImpAnc[t] +
                mutaImpMuc[t] + mutaSecImpAnc[t] + mutaSecImpMuc[t]) +
             predationSecImpAnc * m0 * Exp[-mGrowth * t]) + U * mutaImpAnc[t],


mutaMuc'[t] == mutaMuc[t] *
           (growthMuc - limiting * (ance[t] + mutaMuc[t] + mutaImpAnc[t] + mutaImpMuc[
                t] + mutaSecImpAnc[t] + mutaSecImpMuc[t]) + predationMuc *
              m0 * Exp[-mGrowth * t]) + Ute * ance[t] - U * 4 * mutaMuc[t],

mutaImpMuc'[t] == mutaImpMuc[t] *
           (growthImpMuc - limiting * (ance[t] + mutaMuc[t] + mutaImpAnc[t] +
                mutaImpMuc[t] + mutaSecImpAnc[t] + mutaSecImpMuc[t]) +
             predationImpMuc * m0 * Exp[-mGrowth * t]) +
          U * 4 * mutaMuc[t] - U * 4 * mutaImpMuc[t],

mutaSecImpMuc'[t] == mutaSecImpMuc[t] *
           (growthSecImpMuc - limiting * (ance[t] + mutaMuc[t] + mutaImpAnc[t] +
                mutaImpMuc[t] + mutaSecImpAnc[t] + mutaSecImpMuc[t]) +
             predationSecImpMuc * m0 * Exp[-mGrowth * t]) + U * 4 * mutaImpMuc[t],
```

```
ance[0] == ancestral, mutaMuc[0] == mutantMuc,
      mutaImpAnc[0] == mutantImpAnc, mutaImpMuc[0] == mutantImpMuc,
      mutaSecImpAnc[0] == mutantSecImpAnc, mutaSecImpMuc[0] == mutantSecImpMuc}

, {ance, mutaMuc, mutaImpAnc, mutaImpMuc, mutaSecImpAnc, mutaSecImpMuc}
, {t, Daytime}];

ancestral = ance[Daytime] /. s;
   mutantMuc = mutaMuc[Daytime] /. s; mutantImpAnc = mutaImpAnc[Daytime] /. s;
   mutantImpMuc = mutaImpMuc[Daytime] /. s; mutantSecImpMuc =
    mutaSecImpMuc[Daytime] /. s; mutantSecImpAnc = mutaSecImpAnc[Daytime] /. s;

freqMuc = mutantMuc[[1]] / (mutantMuc[[1]] + ancestral[[1]] + mutantImpAnc[[1]] +
       mutantImpMuc[[1]] + mutantSecImpAnc[[1]] + mutantSecImpMuc[[1]]);
freqImpAnc = mutantImpAnc[[1]] / (mutantMuc[[1]] + ancestral[[1]] +
       mutantImpAnc[[1]] + mutantImpMuc[[1]] +
       mutantSecImpAnc[[1]] + mutantSecImpMuc[[1]]);
freqImpMuc = mutantImpMuc[[1]] / (mutantMuc[[1]] + ancestral[[1]] +
       mutantImpAnc[[1]] + mutantImpMuc[[1]] +
       mutantSecImpAnc[[1]] + mutantSecImpMuc[[1]]);
freqSecImpAnc = mutantSecImpAnc[[1]] / (mutantMuc[[1]] +
       ancestral[[1]] + mutantImpAnc[[1]] + mutantImpMuc[[1]] +
       mutantSecImpAnc[[1]] + mutantSecImpMuc[[1]]);
freqSecImpMuc = mutantSecImpMuc[[1]] / (mutantMuc[[1]] +
       ancestral[[1]] + mutantImpAnc[[1]] + mutantImpMuc[[1]] +
       mutantSecImpAnc[[1]] + mutantSecImpMuc[[1]]);

freqAnc = 1 - (freqMuc + freqImpAnc + freqImpMuc + freqSecImpAnc + freqSecImpMuc);

mutantMuc = Floor[freqMuc * 10^6]; mutantImpAnc = Floor[freqImpAnc * 10^6];
   mutantImpMuc = Floor[freqImpMuc * 10^6];
   mutantSecImpMuc = Floor[freqSecImpMuc * 10^6]; mutantSecImpAnc =
    Floor[freqSecImpAnc * 10^6]; ancestral = Floor[freqAnc * 10^6];

If[i == 1,
impAncOverDays = Append[impAncOverDays, 0];
mucOverDays = Append[mucOverDays, 0];
impMucOverDays = Append[impMucOverDays, 0];
secImpMucOverDays = Append[secImpMucOverDays, 0];
secImpAncOverDays = Append[secImpAncOverDays, 0];
ancestralOverDays = Append[ancestralOverDays, 1];


mucoidOverDays = Append[mucoidOverDays, (freqMuc + freqImpMuc + freqSecImpMuc)];
nonmucoidOverDays =
      Append[nonmucoidOverDays, (freqAnc + freqImpAnc + freqSecImpAnc)];
]

If[Count[assayedDays, i] > 0,
impAncOverDays = Append[impAncOverDays, freqImpAnc];
mucOverDays = Append[mucOverDays, freqMuc];
impMucOverDays = Append[impMucOverDays, freqImpMuc];
secImpMucOverDays = Append[secImpMucOverDays, freqSecImpMuc];
secImpAncOverDays = Append[secImpAncOverDays, freqSecImpAnc];
```

```
ancestralOverDays = Append[ancestralOverDays, ancestral / 10^6];

mucoidOverDays = Append[mucoidOverDays, (freqMuc + freqImpMuc + freqSecImpMuc)];
nonmucoidOverDays =
      Append[nonmucoidOverDays, (freqAnc + freqImpAnc + freqSecImpAnc)];
]
];
results = Append[results, nonmucoidOverDays];
 results = Append[results, mucoidOverDays];

results = Append[results, ancestralOverDays];
results = Append[results, mucOverDays];
results = Append[results, impMucOverDays];
results = Append[results, impAncOverDays];

results = Append[results, secImpMucOverDays];
results = Append[results, secImpAncOverDays];

results]


EvolveFunctionM4[U_?NumericQ,
  Ute_?NumericQ, Utelon_?NumericQ, exciseLon_?NumericQ,
factorGrowthIA_?NumericQ, factorGrowthFirstMuc_?NumericQ,
  factorGrowthLon_?NumericQ, factorGrowthImprovLon_?NumericQ,
  factorGrowthYiaw_?NumericQ, factorGrowthSecImpAnc_?NumericQ,
factorPredationFirstMuc_?NumericQ, factorPredationLon_?NumericQ,
  factorPredationYiaw_?NumericQ] :=

Module[{results = {}, mucoidOverDays = {}, nonmucoidOverDays = {},
   ancestralOverDays = {}, improvAncestralOverDays = {},
   secImprovAncestralOverDays = {}, muc1OverDays = {},
   muc2OverDays = {}, muc3OverDays = {}, muc4OverDays = {},
i, t, assayedDays,
Daytime = 24, Exptime = 31, limiting = 10^-8,
   bGrowthBase = 2.3, predationBase = -3.7 * 10^-6, m0 = 10^6,
   mGrowth = 0.1, ancestral = 10^6, ancestar = 0, ancestartwo = 0,
   muc1yrff = 0, muc2lon = 0, muc3lonyiaw = 0, muc4yrffyiaw = 0,
Utelon2, ance, improvedAnce, secImprovedAnce, firstMuc,
   lonMuc, improvedLonMuc, improvedMuc, growthImprovAnce,
   growthFirstMuc, growthLonMuc, growthImprovLonMuc, growthYiaw,
   growthSecImprovAnce, predationSecImprovAnce, predationImprovAnce,
   predationFirstMuc, predationLonMuc, predationImprovLonMuc,
   predationYiaw, allIndividuals, freqAnc, freqImpAncestral,
   freqMuc1, freqMuc2, freqMuc3, freqMuc4, freqAnceTwo},


(*Ancestral*)
(*U -> Generates Improved Ancestrals*)

(*Improved ancestral*)
growthImprovAnce = bGrowthBase * factorGrowthIA;
  (*ADVANTAGE Factor for growth should be between 1 and 1.1*)
predationImprovAnce = predationBase;
```

```
  (*FIXED Should not change from ancestral*)

(*Improved ancestral2*)
growthSecImprovAnce = growthImprovAnce * factorGrowthSecImpAnc;
  (*ADVANTAGE Factor for growth should be between 1 and 1.1*)
predationSecImprovAnce = predationBase;
  (*FIXED Should not change from ancestral*)



(*First Mucoid (yrfF) appearing, derived from ancestral*)
growthFirstMuc = bGrowthBase * factorGrowthFirstMuc;
  (*COST Factor for growth should be between 0.9 and 1*)
predationFirstMuc = predationBase * factorPredationFirstMuc;
  (*ADVANTAGE Factor for predation should be between 0.8 and 1*)
(*Ute -> Generates lon mucoid OR yiaw*)


(*Improved LON Mucoid, derived from lon mucoid*)
growthImprovLonMuc = growthFirstMuc * factorGrowthImprovLon;
  (*ADVANTAGE Factor for growth should be between 1 and 1.1*)
predationImprovLonMuc = predationFirstMuc;
  (*FIXED Should not change from lon mucoid*)
(*exciseLon -> Can generate yiaw*)

(*yiaw Mucoid,
  derived either from first mucoid (yrfF) or improved Lon Mucoid*)
growthYiaw = growthImprovLonMuc * factorGrowthYiaw;
  (*ADVANTAGE Factor for growth should be between 1 and 1.1*)
predationYiaw = predationImprovLonMuc * factorPredationYiaw;
  (*COST relative to lon; Factor for predation should be between ???*)
(*OR predationYiaw = predationFirstMuc*factorPredationYiaw;
  (*ADVANTAGE relative to first mucoid;
  Factor for predation should be between ???*)*)


assayedDays = {0, 2, 4, 5, 8, 10, 12, 14, 16, 18, 20, 22, 24, 25, 26, 28, 30};


For [i = 1, i < Exptime, i++,
s = NDSolve[
{

ance'[t] ==
       ance[t] * (bGrowthBase - limiting * (ance[t] + improvedAnce[t] + firstMuc[
                t] + improvedLonMuc[t] + improvedMuc[t] + secImprovedAnce[t]) +
            predationBase * m0 * Exp[-mGrowth * t]) - (U (*generates improved
             Ancestral*) + Ute (*generates first mucoid*)) * ance[t],

improvedAnce'[t] == improvedAnce[t] *
          (growthImprovAnce - limiting * (ance[t] + improvedAnce[t] + firstMuc[t] +
               improvedLonMuc[t] + improvedMuc[t] + secImprovedAnce[t]) +
            predationBase * m0 * Exp[-mGrowth * t]) + U
          (*originates from ancestral*) * ance[t] -
         4 * U (*generates improved ancestral 2*) * improvedAnce[t],
```

```
secImprovedAnce'[t] ==
        secImprovedAnce[t] * (growthSecImprovAnce - limiting * (ance[t] +
                improvedAnce[t] + firstMuc[t] + improvedLonMuc[t] + improvedMuc[
                 t] + secImprovedAnce[t]) + predationBase * m0 * Exp[-mGrowth * t]) +
         4 * U (*originates from improved ancestral*) * improvedAnce[t],




firstMuc'[t] == (*yrfF*)
        firstMuc[t] * (growthFirstMuc - limiting * (ance[t] + improvedAnce[t] +
                firstMuc[t] + improvedLonMuc[t] + improvedMuc[t] +
                secImprovedAnce[t]) + predationFirstMuc * m0 * Exp[-mGrowth * t]) +
         Ute (*originates from ancestral*) * ance[t] - Utelon * firstMuc[t],

improvedLonMuc'[t] == (*yrfF + lon + yiaW*)improvedLonMuc[t] *
          (growthImprovLonMuc - limiting * (ance[t] + improvedAnce[t] + firstMuc[
                 t] + improvedLonMuc[t] + improvedMuc[t] + secImprovedAnce[t]) +
             predationImprovLonMuc * m0 * Exp[-mGrowth * t]) +
         Utelon * firstMuc[t] - U * improvedLonMuc[t],

improvedMuc'[t] == (*yrfF + yiaW*)
improvedMuc[t] *
          (growthYiaw - limiting * (ance[t] + improvedAnce[t] + firstMuc[t] +
                improvedLonMuc[t] + improvedMuc[t] + secImprovedAnce[t]) +
             predationYiaw * m0 * Exp[-mGrowth * t]) + U * improvedLonMuc[t],

ance[0] == ancestral, improvedAnce[0] == ancestar,
      firstMuc[0] == muc1yrff, improvedLonMuc[0] == muc3lonyiaw,
      improvedMuc[0] == muc4yrffyiaw, secImprovedAnce[0] == ancestartwo}

, {ance, improvedAnce, firstMuc, improvedLonMuc, improvedMuc, secImprovedAnce}
, {t, Daytime}];

ancestral = ance[Daytime] /. s; ancestar = improvedAnce[Daytime] /. s;
   muc1yrff = firstMuc[Daytime] /. s; muc3lonyiaw = improvedLonMuc[Daytime] /. s;
   muc4yrffyiaw = improvedMuc[Daytime] /. s;
   ancestartwo = secImprovedAnce[Daytime] /. s;


allIndividuals = (ancestral[[1]] + ancestar[[1]] + muc1yrff[[1]] +
      muc3lonyiaw[[1]] + muc4yrffyiaw[[1]] + ancestartwo[[1]]);


freqImpAncestral = ancestar[[1]] / allIndividuals;
freqMuc1 = muc1yrff[[1]] / allIndividuals;
freqMuc3 = muc3lonyiaw[[1]] / allIndividuals;
freqMuc4 = muc4yrffyiaw[[1]] / allIndividuals;
freqAnceTwo = ancestartwo[[1]] / allIndividuals;
freqAnc = 1 - (freqImpAncestral + freqMuc1 + freqMuc3 + freqMuc4 + freqAnceTwo);

(*Simulate Bottleneck -
    population size of an haplotype should be at least 10^6*)
```

```
ancestar = Floor[freqImpAncestral * 10^6]; muc1yrff = Floor[freqMuc1 * 10^6];
muc3lonyiaw = Floor[freqMuc3 * 10^6];
   muc4yrffyiaw = Floor[freqMuc4 * 10^6]; ancestral = Floor[freqAnc * 10^6];
ancestartwo = Floor[freqAnceTwo * 10^6];

If[i == 1,
improvAncestralOverDays = Append[improvAncestralOverDays, 0];
muc1OverDays = Append[muc1OverDays, 0];
muc3OverDays = Append[muc3OverDays, 0];
muc4OverDays = Append[muc4OverDays, 0];

secImprovAncestralOverDays = Append[secImprovAncestralOverDays, 0];
ancestralOverDays = Append[ancestralOverDays, 1];

mucoidOverDays = Append[mucoidOverDays, 0];
nonmucoidOverDays = Append[nonmucoidOverDays, 1];
]


If[Count[assayedDays, i] > 0,
improvAncestralOverDays =
      Append[improvAncestralOverDays, freqImpAncestral];
muc1OverDays = Append[muc1OverDays, freqMuc1];
muc3OverDays = Append[muc3OverDays, freqMuc3];
muc4OverDays = Append[muc4OverDays, freqMuc4];

secImprovAncestralOverDays = Append[secImprovAncestralOverDays, freqAnceTwo];

ancestralOverDays = Append[ancestralOverDays, ancestral / 10^6];

mucoidOverDays = Append[mucoidOverDays, (freqMuc1 + freqMuc3 + freqMuc4)];
nonmucoidOverDays =
      Append[nonmucoidOverDays, (freqAnc + freqImpAncestral + freqAnceTwo)];
]
];
results = Append[results, nonmucoidOverDays];
 results = Append[results, mucoidOverDays];
results = Append[results, ancestralOverDays];
results = Append[results, improvAncestralOverDays];
results = Append[results, muc1OverDays];
results = Append[results, muc3OverDays];
results = Append[results, muc4OverDays];

results = Append[results, secImprovAncestralOverDays];
results]



EvolveFunctionM5[U_?NumericQ, Ute_?NumericQ,
factorGrowthM1_?NumericQ, factorGrowthM2_?NumericQ,
  factorGrowthM3_?NumericQ, factorGrowthM4_?NumericQ,
factorPredationM1_?NumericQ, factorPredationM3_?NumericQ] :=

Module[{results = {}, mucoidOverDays = {},
   nonmucoidOverDays = {}, ancestralOverDays = {}, firstMucOverDays = {},
```

```
    impAncOverDays = {}, secMucOverDays = {}, secImpAncOverDays = {},
i, t, assayedDays = {},
Daytime = 24, Exptime = 31, limiting = 10^-8, bGrowthBase = 2.3,
    predationBase = -3.7 * 10^-6, m0 = 10^6, mGrowth = 0.1, ancestral = 10^6,
    mutant1 = 0, mutant2 = 0, mutant3 = 0, mutant4 = 0, mutant5 = 0,
ance, firstMuc, impAnc, secMuc, secImpAnc, thrdMuc, growthMut1,
    growthMut2, growthMut3, growthMut4, growthMut5, predationMut1,
    predationMut2, predationMut3, predationMut4, predationMut5, freqAnc,
    freqFirstMuc, freqImpAnc, freqSecMuc, freqSecImpAnc, freqThrdMuc},

(*Mucoid derived from ancestral*)
growthMut1 = bGrowthBase * factorGrowthM1;
   (*COST Factor should be between 0.9 and 1*)
predationMut1 = predationBase * factorPredationM1;
   (*ADVANTAGE Factor should be between 0.8 and 1*)

(*Improved ancestral*)
growthMut2 = bGrowthBase * factorGrowthM2;
   (*ADVANTAGE Factor should be between 1 and 1.1*)
predationMut2 = predationBase; (*FIXED Should not change from ancestral*)

(*Improved mucoid*)
growthMut3 = growthMut1 * factorGrowthM3;
   (*ADVANTAGE Factor (growthM3) should be between 1 and 1.1*)
predationMut3 = predationMut1 * factorPredationM3;
   (*FIXED Should not change from previous mucoid*)

(*Improved ancestral2*)
growthMut4 = growthMut2 * factorGrowthM4;
   (*ADVANTAGE Factor should be between 1 and 1.1*)
predationMut4 = predationBase; (*FIXED Should not change from ancestral*)


assayedDays = {0, 2, 4, 5, 8, 10, 12, 14, 16, 18, 20, 22, 24, 25, 26, 28, 30};

(*Print[U,",",Ute,",",Utelon,",",growthMut1,",",growthMut2,",",growthMut3,
    ",",predationMut1,",",predationMut2,",",predationMut3 ]*)

For [i = 1, i < Exptime, i++,
s = NDSolve[
{
ance'[t] == ance[t] * (bGrowthBase - limiting *
                (ance[t] + firstMuc[t] + impAnc[t] + secMuc[t] + secImpAnc[t]) +
              predationBase * m0 * Exp[-mGrowth * t]) - (U + Ute) * ance[t],

impAnc'[t] == impAnc[t] * (growthMut2 - limiting *
                (ance[t] + firstMuc[t] + impAnc[t] + secMuc[t] + secImpAnc[t]) +
              predationMut2 * m0 * Exp[-mGrowth * t]) + U * ance[t] - U * impAnc[t],

secImpAnc'[t] == secImpAnc[t] * (growthMut4 - limiting *
                (ance[t] + firstMuc[t] + impAnc[t] + secMuc[t] + secImpAnc[t]) +
              predationMut4 * m0 * Exp[-mGrowth * t]) + U * impAnc[t],


firstMuc'[t] == firstMuc[t] * (growthMut1 - limiting * (ance[t] + firstMuc[t] +
```

```
                impAnc[t] + secMuc[t] + secImpAnc[t]) + predationMut1 *
              m0 * Exp[-mGrowth * t]) + Ute * ance[t] - U / 4 * firstMuc[t],

secMuc'[t] == secMuc[t] * (growthMut3 - limiting *
              (ance[t] + firstMuc[t] + impAnc[t] + secMuc[t] + secImpAnc[t]) +
             predationMut3 * m0 * Exp[-mGrowth * t]) + U / 4 * firstMuc[t],



ance[0] == ancestral, firstMuc[0] == mutant1,
      impAnc[0] == mutant2, secMuc[0] == mutant3, secImpAnc[0] == mutant4}

, {ance, firstMuc, impAnc, secMuc, secImpAnc}
, {t, Daytime}];

ancestral = ance[Daytime] /. s;
   mutant1 = firstMuc[Daytime] /. s; mutant2 = impAnc[Daytime] /. s;
   mutant3 = secMuc[Daytime] /. s; mutant4 = secImpAnc[Daytime] /. s;

freqFirstMuc = mutant1[[1]] / (mutant1[[1]] +
       ancestral[[1]] + mutant2[[1]] + mutant3[[1]] + mutant4[[1]]);
freqImpAnc = mutant2[[1]] / (mutant1[[1]] + ancestral[[1]] +
       mutant2[[1]] + mutant3[[1]] + mutant4[[1]]);
freqSecMuc = mutant3[[1]] / (mutant1[[1]] + ancestral[[1]] +
       mutant2[[1]] + mutant3[[1]] + mutant4[[1]]);
freqSecImpAnc = mutant4[[1]] / (mutant1[[1]] + ancestral[[1]] +
       mutant2[[1]] + mutant3[[1]] + mutant4[[1]]);
freqAnc = 1 - (freqFirstMuc + freqImpAnc + freqSecMuc + freqSecImpAnc);

mutant1 = Floor[freqFirstMuc * 10^6];
   mutant2 = Floor[freqImpAnc * 10^6]; mutant3 = Floor[freqSecMuc * 10^6];
   mutant4 = Floor[freqSecImpAnc * 10^6]; ancestral = Floor[freqAnc * 10^6];

If[i == 1,
firstMucOverDays = Append[firstMucOverDays, 0];
impAncOverDays = Append[impAncOverDays, 0];
secMucOverDays = Append[secMucOverDays, 0];
secImpAncOverDays = Append[secImpAncOverDays, 0];
ancestralOverDays = Append[ancestralOverDays, 1];

mucoidOverDays = Append[mucoidOverDays, 0];
nonmucoidOverDays = Append[nonmucoidOverDays, 1];
]

If[Count[assayedDays, i] > 0,
firstMucOverDays = Append[firstMucOverDays, freqFirstMuc];
impAncOverDays = Append[impAncOverDays, freqImpAnc];
secMucOverDays = Append[secMucOverDays, freqSecMuc];
secImpAncOverDays = Append[secImpAncOverDays, freqSecImpAnc];
ancestralOverDays = Append[ancestralOverDays, ancestral / 10^6];

mucoidOverDays = Append[mucoidOverDays, (freqFirstMuc + freqSecMuc)];
nonmucoidOverDays =
      Append[nonmucoidOverDays, (freqAnc + freqImpAnc + freqSecImpAnc)];
]
```

```
];
results = Append[results, nonmucoidOverDays];
  results = Append[results, mucoidOverDays];
results = Append[results, ancestralOverDays];
results = Append[results, firstMucOverDays];
results = Append[results, impAncOverDays];
results = Append[results, secMucOverDays];
results = Append[results, secImpAncOverDays];
results]



EvolveFunctionM6[U_?NumericQ, Ute_?NumericQ, factorGrowthImpAnc_?NumericQ,
   factorGrowthMuc_?NumericQ, factorGrowthImpMuc_?NumericQ,
factorPredationMuc_?NumericQ] :=

Module[{results = {}, mucoidOverDays = {},
    nonmucoidOverDays = {}, ancestralOverDays = {},
    impAncOverDays = {}, mucOverDays = {}, impMucOverDays = {},
i, t, assayedDays,
Daytime = 24, Exptime = 31, limiting = 10^-8, bGrowthBase = 2.3,
    predationBase = -3.7 * 10^-6, m0 = 10^6, mGrowth = 0.1,
    ancestral = 10^6, mutantMuc = 0, mutantImpAnc = 0, mutantImpMuc = 0,
ance, mutaMuc, mutaImpAnc, mutaImpMuc, growthMuc, growthImpAnc,
    growthImpMuc, predationMuc, predationImpAnc,
    predationImpMuc, freqAnc, freqMuc, freqImpAnc, freqImpMuc},

(*Mucoid derived from ancestral*)
growthMuc = bGrowthBase * factorGrowthMuc;
   (*COST Factor should be between 0.9 and 1*)
predationMuc = predationBase * factorPredationMuc;
   (*ADVANTAGE Factor should be between 0.8 and 1*)

(*Improved ancestral*)
growthImpAnc = bGrowthBase * factorGrowthImpAnc;
   (*ADVANTAGE Factor should be between 1 and 1.1*)
predationImpAnc = predationBase; (*FIXED Should not change from ancestral*)

(*Improved Mucoid*)
growthImpMuc = growthMuc * factorGrowthImpMuc;
   (*ADVANTAGE Factor should be between 1 and 1.1*)
predationImpMuc = predationMuc;
   (*FIXED Should not change from first mucoid*)

assayedDays = {0, 2, 4, 5, 8, 10, 12, 14, 16, 18, 20, 22, 24, 25, 26, 28, 30};

For [i = 1, i < Exptime, i++,
s = NDSolve[
{
ance'[t] == ance[t] * (bGrowthBase -
            limiting * (ance[t] + mutaMuc[t] + mutaImpAnc[t] + mutaImpMuc[t]) +
            predationBase * m0 * Exp[-mGrowth * t]) - (U + Ute) * ance[t],

mutaImpAnc'[t] == mutaImpAnc[t] * (growthImpAnc -
            limiting * (ance[t] + mutaMuc[t] + mutaImpAnc[t] + mutaImpMuc[t]) +
```

```
            predationImpAnc * m0 * Exp[-mGrowth * t]) + U * ance[t],

mutaMuc'[t] == mutaMuc[t] * (growthMuc - limiting *
              (ance[t] + mutaMuc[t] + mutaImpAnc[t] + mutaImpMuc[t]) + predationMuc *
              m0 * Exp[-mGrowth * t]) + Ute * ance[t] - U * 4 * mutaMuc[t],

mutaImpMuc'[t] == mutaImpMuc[t] * (growthImpMuc -
            limiting * (ance[t] + mutaMuc[t] + mutaImpAnc[t] + mutaImpMuc[t]) +
            predationImpMuc * m0 * Exp[-mGrowth * t]) + U * 4 * mutaMuc[t],

ance[0] == ancestral, mutaMuc[0] == mutantMuc,
      mutaImpAnc[0] == mutantImpAnc, mutaImpMuc[0] == mutantImpMuc}

, {ance, mutaMuc, mutaImpAnc, mutaImpMuc}
, {t, Daytime}];

ancestral = ance[Daytime] /. s; mutantMuc = mutaMuc[Daytime] /. s; mutantImpAnc =
    mutaImpAnc[Daytime] /. s; mutantImpMuc = mutaImpMuc[Daytime] /. s;

freqMuc = mutantMuc[[1]] /
     (mutantMuc[[1]] + ancestral[[1]] + mutantImpAnc[[1]] + mutantImpMuc[[1]]);
freqImpAnc = mutantImpAnc[[1]] / (mutantMuc[[1]] + ancestral[[1]] +
       mutantImpAnc[[1]] + mutantImpMuc[[1]]);
freqImpMuc = mutantImpMuc[[1]] / (mutantMuc[[1]] + ancestral[[1]] +
       mutantImpAnc[[1]] + mutantImpMuc[[1]]);
freqAnc = 1 - (freqMuc + freqImpAnc + freqImpMuc);

mutantMuc = Floor[freqMuc * 10^6]; mutantImpAnc = Floor[freqImpAnc * 10^6];
   mutantImpMuc = Floor[freqImpMuc * 10^6]; ancestral = Floor[freqAnc * 10^6];

If[i == 1,
impAncOverDays = Append[impAncOverDays, 0];
mucOverDays = Append[mucOverDays, 0];
impMucOverDays = Append[impMucOverDays, 0];
ancestralOverDays = Append[ancestralOverDays, 1];

mucoidOverDays = Append[mucoidOverDays, (freqMuc + freqImpMuc)];
nonmucoidOverDays = Append[nonmucoidOverDays, (freqAnc + freqImpAnc)];
]

If[Count[assayedDays, i] > 0,
impAncOverDays = Append[impAncOverDays, freqImpAnc];
mucOverDays = Append[mucOverDays, freqMuc];
impMucOverDays = Append[impMucOverDays, freqImpMuc];
ancestralOverDays = Append[ancestralOverDays, ancestral / 10^6];

mucoidOverDays = Append[mucoidOverDays, (freqMuc + freqImpMuc)];
nonmucoidOverDays = Append[nonmucoidOverDays, (freqAnc + freqImpAnc)];
]
];
results = Append[results, nonmucoidOverDays];
 results = Append[results, mucoidOverDays];

results = Append[results, ancestralOverDays];
results = Append[results, mucOverDays];
```

```
results = Append[results, impMucOverDays];
results = Append[results, impAncOverDays];
results]




(*LINE A*)
ExperimentalDataA = Import[
   "/Users/jorgeabsousa/Dropbox/PhD Lab/###PROJECTS###/MucoidDynamics/Mucoid
     Dynamics/MucDynamics/M1Data.csv", "CSV"];
ExperimentalDataA;
ExperimentalDataA[[1]] = Delete[ExperimentalDataA[[1]], 1];
ExperimentalDataA[[2]] = Delete[ExperimentalDataA[[2]], 1];
ExperimentalDataA[[3]] = Delete[ExperimentalDataA[[3]], 1];
ExperimentalDataA[[4]] = Delete[ExperimentalDataA[[4]], 1];
ExperimentalDataA[[5]] = Delete[ExperimentalDataA[[5]], 1];
ExperimentalDataA[[6]] = Delete[ExperimentalDataA[[6]], 1];

sU = 3 * 10^-7;
sUte = 4 * 10^-7;
sfacGrowthImpAnc = 1.03; (*Ancestral -> Improved Ancestral*)
sfacGrowthMuc = 0.946; (*Ancestral -> Mucoid*)
sfacGrowthImpMuc = 1.019; (*Mucoid -> Improved Mucoid*)
sfacPredMuc = 0.874; (*Ancestral -> Mucoid*)
r121A = EvolveFunctionM1[sU, sUte, sfacGrowthImpAnc,
  sfacGrowthMuc, sfacGrowthImpMuc, sfacPredMuc];

PlotFunction[ExperimentalDataA, r121A]

(*LINE B*)
ExperimentalDataB = Import[
   "/Users/jorgeabsousa/Dropbox/PhD Lab/###PROJECTS###/MucoidDynamics/Mucoid
     Dynamics/MucDynamics/M2Data.csv", "CSV"];
ExperimentalDataB;
ExperimentalDataB[[1]] = Delete[ExperimentalDataB[[1]], 1];
ExperimentalDataB[[2]] = Delete[ExperimentalDataB[[2]], 1];
ExperimentalDataB[[3]] = Delete[ExperimentalDataB[[3]], 1];
ExperimentalDataB[[4]] = Delete[ExperimentalDataB[[4]], 1];
ExperimentalDataB[[5]] = Delete[ExperimentalDataB[[5]], 1];
ExperimentalDataB[[6]] = Delete[ExperimentalDataB[[6]], 1];


(*Test function with parameters*)
sU = 9.6 * 10^-8;
sUte = 4.45 * 10^-7;
sfacGrowthImpAnc = 1.04; (*Ancestral -> Improved Ancestral*)
sfacGrowthMuc = 0.9614; (*Ancestral -> Mucoid*)
sfacGrowthImpMuc = 1.02; (*Mucoid -> Improved Mucoid*)
sfacPredMuc = 0.864; (*Ancestral -> Mucoid*)
r121B = EvolveFunctionM2[sU, sUte, sfacGrowthImpAnc,
  sfacGrowthMuc, sfacGrowthImpMuc, sfacPredMuc];

PlotFunction[ExperimentalDataB, r121B]
```

```
(*LINE C*)
ExperimentalDataC = Import[
   "/Users/jorgeabsousa/Dropbox/PhD Lab/###PROJECTS###/MucoidDynamics/Mucoid
      Dynamics/MucDynamics/M3Data.csv", "CSV"];
ExperimentalDataC;
ExperimentalDataC[[1]] = Delete[ExperimentalDataC[[1]], 1];
ExperimentalDataC[[2]] = Delete[ExperimentalDataC[[2]], 1];
ExperimentalDataC[[3]] = Delete[ExperimentalDataC[[3]], 1];
ExperimentalDataC[[4]] = Delete[ExperimentalDataC[[4]], 1];
ExperimentalDataC[[5]] = Delete[ExperimentalDataC[[5]], 1];
ExperimentalDataC[[6]] = Delete[ExperimentalDataC[[6]], 1];


sU = 4.3 * 10^-8;
sUte = 4 * 10^-7;
sfacGrowthImpAnc = 1.0265; (*Ancestral -> Improved Ancestral*)
sfacGrowthMuc = 0.95; (*Ancestral -> Mucoid*)
sfacGrowthImpMuc = 1.04; (*Mucoid -> Improved Mucoid*)
sfacPredMuc = 0.877; (*Ancestral -> Mucoid*)
sfacGrowthSecImpMuc = 0.945; (*Improved Mucoid -> Improved Mucoid2*)
sfacGrowthSecImpAnc = 1.0475; (*ImprovedAncestral -> Improved Ancestral2*)

r121C = EvolveFunctionM3[sU, sUte, sfacGrowthImpAnc, sfacGrowthMuc,
  sfacGrowthImpMuc, sfacGrowthSecImpMuc, sfacGrowthSecImpAnc, sfacPredMuc];

PlotFunction[ExperimentalDataC, r121C]
```

```
(*LINE D*)
ExperimentalDataD = Import[
   "/Users/jorgeabsousa/Dropbox/PhD Lab/###PROJECTS###/MucoidDynamics/Mucoid
     Dynamics/MucDynamics/M4Data.csv", "CSV"];
ExperimentalDataD;
ExperimentalDataD[[1]] = Delete[ExperimentalDataD[[1]], 1];
ExperimentalDataD[[2]] = Delete[ExperimentalDataD[[2]], 1];
ExperimentalDataD[[3]] = Delete[ExperimentalDataD[[3]], 1];
ExperimentalDataD[[4]] = Delete[ExperimentalDataD[[4]], 1];
ExperimentalDataD[[5]] = Delete[ExperimentalDataD[[5]], 1];
ExperimentalDataD[[6]] = Delete[ExperimentalDataD[[6]], 1];

U = 1 * 10^-8; Ute = 10 * 10^-7; Utelon = 5 * 10^-7; exciseLon = 2 * 10^-5;

(*Ancestral -> Improved Ancestral*)
facGrowthIA = 1.048;  (*More growth*)
(*Predation same as ancstral*)

(*Ancestral -> First Mucoid (yrfF)*)
facGrowthFirstMuc = 0.956;  (*Less growth*)
facPredationFirstMuc = 0.864; (*Less predated*)

(*First Mucoid (yrfF) -> Lon Mucoid (yrfF + lon)*)
facGrowthLonMuc = 0.905;  (*Much less growth*)
facPredationLonMuc = 0.79; (*Much less predated*)

(*Improved Ancestral -> Improved Ancestral2*)
facGrowthSecImpAnc = 1.035;  (*More growth*)

(*Lon Mucoid (yrfF + lon)-> Improved Lon Mucoid (yrfF + lon + yiaW)*)
facGrowthImprovedLon = 1.032;  (*More growth*)
(*predation same as lon*)

(*Improved Lon Mucoid (yrfF + lon) OR First Mucoid (yrfF)->
 Improved Mucoid (yrfF + yiaW)*)
facGrowthImprovedMuc = 1.;
(*More growth (relative to yrfF or yrfF + lon + yiaW*)
facPredationImprovedMuc = 0.8825;
(*More predated (relative to yrfF + lon + yiaW)*)


r121D = EvolveFunctionM4[U, Ute, Utelon,
  exciseLon, facGrowthIA, facGrowthFirstMuc, facGrowthLonMuc,
  facGrowthImprovedLon, facGrowthImprovedMuc, facGrowthSecImpAnc,
  facPredationFirstMuc, facPredationLonMuc, facPredationImprovedMuc];

PlotFunction[ExperimentalDataD, r121D]
```

```
(*LINE E*)
ExperimentalDataE = Import[
   "/Users/jorgeabsousa/Dropbox/PhD Lab/###PROJECTS###/MucoidDynamics/Mucoid
     Dynamics/MucDynamics/M5Data.csv", "CSV"];
ExperimentalDataE;
ExperimentalDataE[[1]] = Delete[ExperimentalDataE[[1]], 1];
ExperimentalDataE[[2]] = Delete[ExperimentalDataE[[2]], 1];
ExperimentalDataE[[3]] = Delete[ExperimentalDataE[[3]], 1];
ExperimentalDataE[[4]] = Delete[ExperimentalDataE[[4]], 1];
ExperimentalDataE[[5]] = Delete[ExperimentalDataE[[5]], 1];
ExperimentalDataE[[6]] = Delete[ExperimentalDataE[[6]], 1];

(*Test function with parameters*)
sU = 4.3 * 10^-8;
sUte = 6.94941 * 10^-7;
sfacGrowth1 = 0.94816; (*Ancestral -> Mucoid*)
sfacGrowth2 = 1.02; (*Ancestral -> Improved Ancestral*)
sfacGrowth3 = 1.; (*Mucoid -> Improved Mucoid*)
sfacGrowth4 = 1.058; (*Improved Ancestral -> Improved Ancestral2*)
sfacPred1 = 0.8858; (*Ancestral -> Mucoid*)
sfacPred3 = 0.8725; (*Mucoid -> Improved Mucoid*)

r121E = EvolveFunctionM5[sU, sUte, sfacGrowth1,
  sfacGrowth2, sfacGrowth3, sfacGrowth4, sfacPred1, sfacPred3];


PlotFunction[ExperimentalDataE, r121E]

(*LINE F*)
ExperimentalDataF = Import[
   "/Users/jorgeabsousa/Dropbox/PhD Lab/###PROJECTS###/MucoidDynamics/Mucoid
     Dynamics/MucDynamics/M6Data.csv", "CSV"];
ExperimentalDataF;
ExperimentalDataF[[1]] = Delete[ExperimentalDataF[[1]], 1];
ExperimentalDataF[[2]] = Delete[ExperimentalDataF[[2]], 1];
ExperimentalDataF[[3]] = Delete[ExperimentalDataF[[3]], 1];
ExperimentalDataF[[4]] = Delete[ExperimentalDataF[[4]], 1];
ExperimentalDataF[[5]] = Delete[ExperimentalDataF[[5]], 1];
ExperimentalDataF[[6]] = Delete[ExperimentalDataF[[6]], 1];


(*Test function with parameters*)
sU = 30 * 10^-8;
sUte = 4 * 10^-7;
sfacGrowthImpAnc = 1.032; (*Ancestral -> Improved Ancestral*)
sfacGrowthMuc = 0.946; (*Ancestral -> Mucoid*)
sfacGrowthImpMuc = 1.0225; (*Mucoid -> Improved Mucoid*)
sfacPredMuc = 0.872; (*Ancestral -> Mucoid*)
r121F = EvolveFunctionM6[sU, sUte, sfacGrowthImpAnc,
  sfacGrowthMuc, sfacGrowthImpMuc, sfacPredMuc];

PlotFunction[ExperimentalDataF, r121F]

(*JOINT DYNAMICS*)

Needs["PlotLegends`"]
```

```
JoinPlotFunctionFull[ExperDataA_, ExperDataB_,
   ExperDataC_, ExperDataD_, ExperDataE_, ExperDataF_, SimDataA_,
   SimDataB_, SimDataC_, SimDataD_, SimDataE_, SimDataF_] := Table[{

days = {0, 2, 4, 5, 8, 10, 12, 14, 16, 18, 20, 22, 24, 25, 26, 28, 30};

ListLinePlot[{
(*Table[{days[[i]],ExperDataA[[3]][[i]]},{i, 1, 17}],
Table[{days[[i]],ExperDataB[[3]][[i]]},{i, 1, 17}],
Table[{days[[i]],ExperDataC[[3]][[i]]},{i, 1, 17}],
Table[{days[[i]],ExperDataD[[3]][[i]]},{i, 1, 17}],
Table[{days[[i]],ExperDataE[[3]][[i]]},{i, 1, 17}],
Table[{days[[i]],ExperDataF[[3]][[i]]},{i, 1, 17}],*)

Table[{days[[i]], SimDataA[[2]][[i]]}, {i, 1, 17}],
Table[{days[[i]], SimDataB[[2]][[i]]}, {i, 1, 17}],
Table[{days[[i]], SimDataC[[2]][[i]]}, {i, 1, 17}],
Table[{days[[i]], SimDataD[[2]][[i]]}, {i, 1, 17}],
Table[{days[[i]], SimDataE[[2]][[i]]}, {i, 1, 17}],
Table[{days[[i]], SimDataF[[2]][[i]]}, {i, 1, 17}]

},
(*Mesh->Full,PlotStyle->{{Black, Dashed},Black, {Red, Dashed}, Red},*)
(*Mesh->Full,PlotStyle->{Black,Red, Gray, Green, Purple,
       Yellow, {Black, Dashed},{Red, Dashed}, {Gray, Dashed},
       {Green, Dashed}, {Purple, Dashed}, {Yellow, Dashed}},*)
PlotStyle -> {Black, Black, Black, Black, Black, Black},
PlotMarkers -> {Automatic, Large}, PlotRange -> {{0, 31}, {-0.02, 1.02}},
     Frame -> {{True, False}, {True, False}},
     FrameLabel -> {"Days", "Frequency"},
     FrameTicks -> {Table[i, {i, 0, 30, 2}], Table[i, {i, 0, 1, 0.1}]},
     FrameStyle -> {{Thick, Thick}, {Thick, Thick}}, BaseStyle ->
      {FontFamily -> "Arial", Bold, FontSize -> 25}, ImageSize -> 1000,
PlotLegend -> {
Style["M1", 30, FontFamily -> "Arial", FontWeight -> "Bold"],
Style["M2", 30, FontFamily -> "Arial", FontWeight -> "Bold"],
Style["M3", 30, FontFamily -> "Arial", FontWeight -> "Bold"],
Style["M4", 30, FontFamily -> "Arial", FontWeight -> "Bold"],
Style["M5", 30, FontFamily -> "Arial", FontWeight -> "Bold"],
Style["M6", 30, FontFamily -> "Arial", FontWeight -> "Bold"]},
LegendPosition -> {1.1, -0.4}, LegendShadow -> None,
     LegendBorder -> None, PlotMarkers -> Automatic]

}

];


JoinPlotFunctionFull[ExperimentalDataA, ExperimentalDataB,
 ExperimentalDataC, ExperimentalDataD, ExperimentalDataE,
 ExperimentalDataF, r121A, r121B, r121C, r121D, r121E, r121F]
```

```
JoinPlotFunctionSeparate[ExperDataA_, ExperDataB_,
   ExperDataC_, ExperDataD_, ExperDataE_, ExperDataF_, SimDataA_,
   SimDataB_, SimDataC_, SimDataD_, SimDataE_, SimDataF_] := Table[{

days = {0, 2, 4, 5, 8, 10, 12, 14, 16, 18, 20, 22, 24, 25, 26, 28, 30};

ListLinePlot[{
Table[{days[[i]], ExperDataA[[3]][[i]]}, {i, 1, 17}],
Table[{days[[i]], ExperDataB[[3]][[i]]}, {i, 1, 17}],
Table[{days[[i]], ExperDataC[[3]][[i]]}, {i, 1, 17}],

Table[{days[[i]], SimDataA[[2]][[i]]}, {i, 1, 17}],
Table[{days[[i]], SimDataB[[2]][[i]]}, {i, 1, 17}],
Table[{days[[i]], SimDataC[[2]][[i]]}, {i, 1, 17}]
},
(*Mesh->Full,PlotStyle->{{Black, Dashed},Black, {Red, Dashed}, Red},*)
(*Mesh->Full,PlotStyle->
      {Black,Red, Gray, {Black, Dashed},{Red, Dashed}, {Gray, Dashed}},*)
Mesh -> Full, PlotStyle -> Automatic,
PlotMarkers -> {Automatic, Medium},
     PlotRange -> {{0, 31}, {-0.02, 1.02}}, Frame ->
      {{True, False}, {True, False}}, FrameLabel -> {"Days", "Frequency"},
     FrameTicks -> {Table[i, {i, 0, 30, 2}], Table[i, {i, 0, 1, 0.1}]},
     FrameStyle -> {{Thick, Thick}, {Thick, Thick}},
     BaseStyle -> {Bold, FontSize -> 18}, ImageSize -> 500],

ListLinePlot[{

Table[{days[[i]], ExperDataD[[3]][[i]]}, {i, 1, 17}],
Table[{days[[i]], ExperDataE[[3]][[i]]}, {i, 1, 17}],
Table[{days[[i]], ExperDataF[[3]][[i]]}, {i, 1, 17}],

Table[{days[[i]], SimDataD[[2]][[i]]}, {i, 1, 17}],
Table[{days[[i]], SimDataE[[2]][[i]]}, {i, 1, 17}],
Table[{days[[i]], SimDataF[[2]][[i]]}, {i, 1, 17}]
},
(*Mesh->Full,PlotStyle->{{Black, Dashed},Black, {Red, Dashed}, Red},*)
Mesh -> Full, PlotStyle ->
      {Black, Red, Gray, {Black, Dashed}, {Red, Dashed}, {Gray, Dashed}},
PlotMarkers -> {Automatic, Medium}, PlotRange -> {{0, 31}, {-0.02, 1.02}},
     Frame -> {{True, False}, {True, False}},
     FrameLabel -> {"Days", "Frequency"},
     FrameTicks -> {Table[i, {i, 0, 30, 2}], Table[i, {i, 0, 1, 0.1}]},
     FrameStyle -> {{Thick, Thick}, {Thick, Thick}},
     BaseStyle -> {Bold, FontSize -> 18}, ImageSize -> 500]

}];

JoinPlotFunctionSeparate[ExperimentalDataA, ExperimentalDataB,
 ExperimentalDataC, ExperimentalDataD, ExperimentalDataE,
 ExperimentalDataF, r121A, r121B, r121C, r121D, r121E, r121F]
```
