# Supplementary material for: The Genetic Basis of Escherichia coli Pathoadaptation to Macrophages
Source: PLoS Pathog. 2013 Dec 12;9(12):e1003802. doi: 10.1371/journal.ppat.1003802 (PMC3861542; doi:10.1371/journal.ppat.1003802)
Supplement: Table S1 — Increased resistance of the SCV clone to aminoglycoside antibiotics. Minimal inhibitory concentration (MIC) of each clone was measured in triplicate by a disc diffusion assay. S indicates sensitive clones and R resistant clones. SCV_M1_D8, SCV_M2_D4, SCV_M3_D5 and MUC_M2_D19, MUC_M3_D19, MUC_M4_D19 clones are shown. (DOC) [file ppat.1003802.s014.doc]

|  | **Mean zone diameter (mm)** | | | | | | |
| --- | --- | --- | --- | --- | --- | --- | --- |
| **Antibiotic (µg)** | **ANC** | **SCV1** | **SCV2** | **SCV3** | **MUC2** | **MUC3** | **MUC4** |
| Kanamycin (30) | 24S | 11R | 12R | 10R | 23S | 20S | 21S |
| Gentamicin (10) | 20S | 10R | 10R | 10R | 16S | 16S | 18S |
| Amikacine (30) | 24S | 12R | 11R | 11R | 20S | 17S | 18S |
| Tobramycin (10) | 18S | 11R | 10R | 10R | 17S | 17S | 17S |
| Netilmycin (10) | 24S | 16S | 15S | 15S | 19S | 17S | 21S |
| Tetracycline (30) | 29S | 33S | 31S | 32S | 30S | 33S | 32S |
| Nalidixic acid (30) | 27S | 34S | 32S | 33S | 34S | 34S | 36S |

Minimal inhibitory concentration (MIC) of each clone was measured in triplicate by a disc diffusion assay. S indicates sensitive clones and R resistant clones. SCV_M1_D8, SCV_M2_D4, SCV_M3_D5 and MUC_M2_D19, MUC_M3_D19, MUC_M4_D19 clones are shown.
