# Supplementary material for: The Genetic Basis of Escherichia coli Pathoadaptation to Macrophages
Source: PLoS Pathog. 2013 Dec 12;9(12):e1003802. doi: 10.1371/journal.ppat.1003802 (PMC3861542; doi:10.1371/journal.ppat.1003802)
Supplement: Table S2 — Primers used in this study. (DOC) [file ppat.1003802.s015.doc]

| **Amplified region** | **Primers (5` - 3`)** | **PCR product (bp)** |
| --- | --- | --- |
| ***fusA*** | F: CAATTACGGCCTGAGCAACG  R: CTGCACCTCGACATCATCGT | 709 |
| ***yegH*** | F: CGGATGATTGAGCGGGTACT  R: ATCTCGCCATCCTTACGCAG | 704 |
| ***folD*** | F: CCCGACTTCTTCACAAGCCT  R: AGAGAGCCCCGTTAGATGAA | 518 |
| ***trkH*** | F: CGTTGGACTACTGGTCATCTT  R: CAGGTAGGAGGCAATCTCGC | 1500 |
| ***wzc*** | F: CGGCTTATGAGCTGGTTTGC  R: AAAGCCCGCAAGTACTGGAA | 883 |
| ***hipA*** | F: ATAGCGATATCCTGCGACCT  R: AATCATGACCGCCAACGACT | 773 |
| ***lon*** | F: GCAATACGGGGATTTCAATG  R: GGAAGACGTCGAAAAAGTGG | 408 |
| ***yrfF*** | F: TTGCTCATCGTGGGATCTATGCTGT  R: TCGGCATTCAGCGCCAGAGA | 578 |
| ***yiaW*** | F: TCACCACACCAGTCACCTGT  R: CCTGGACTATTTTGCACTGGGA | 547 |
| ***potA*** | F: CATCAGCCAGTACGACCTCC  R: CAATTGGCGGGAATTCGCAA | 1069 |
| ***potD*** | F: GCCTTCGGTATGAATCGCGT  R: ATTACTGGTGCTGTCGCTGG | 1314 |
| **IS186B** | acctgaactcgcgaaagcgtggata |  |
| ***nfsA*** | F: GCTGCGGTGGTGGTTATTCT  R: CATCCCGGGACAATATGGCA | 908 |
