# Supplementary material for: The Genetic Basis of Escherichia coli Pathoadaptation to Macrophages
Source: PLoS Pathog. 2013 Dec 12;9(12):e1003802. doi: 10.1371/journal.ppat.1003802 (PMC3861542; doi:10.1371/journal.ppat.1003802)
Supplement: Table S3 — Parameters used in modeling the dynamics of the different haplotypes. Parameters used for the dynamics in figure S9. Cases where more haplotypes were assumed to reproduce the experimental dynamics are marked with *, and the additional parameters are in Table S4. (DOC) [file ppat.1003802.s016.doc]

|  | *U*  (x10^-7^) | *Uis*  (x10^-7^) | *r_m_* | *a_mmuc_* (x10^-6^) | *Uis*’  (x10^-7^) | *r*’ | *a_mb_*  (x10^-6^) | *r_m_*’ | *a_mmuc_*’  (x10^-6^) |
| --- | --- | --- | --- | --- | --- | --- | --- | --- | --- |
| **A** | 3 | 4 | 2.1758 | -3.23 | 12 | 2.369 | -3.7 | 2.2171402 | -3.23 |
| **B** | 0.96 | 4.45 | 2.21122 | -3.2 | 3.84 | 2.392 | -3.7 | 2.2554444 | -3.2 |
| **C*** | 0.43 | 4 | 2.185 | -3.24 | 1.72 | 2.36095 | -3.7 | 2.2724 | -3.24 |
| **D*** | 0.1 | 10 | 2.1988 | -3.2 | 5 | 2.4104 | -3.7 | 2.2691616 | -3.2 |
| **E*** | 0.43 | 6.94 | 2.180768 | -3.28 | 0.1075 | 2.346 | -3.7 | 2.180768 | -2.86 |
| **F** | 3 | 4 | 2.1758 | -3.23 | 12 | 2.3736 | -3.7 | 2.2247555 | -3.23 |
