# Supplementary material for: The Genetic Basis of Escherichia coli Pathoadaptation to Macrophages
Source: PLoS Pathog. 2013 Dec 12;9(12):e1003802. doi: 10.1371/journal.ppat.1003802 (PMC3861542; doi:10.1371/journal.ppat.1003802)
Supplement: Table S4 — Parameters for the additional haplotypes for the modeled dynamics. Parameters in the additional haplotypes required to obtain the dynamics in Figure S9. (DOC) [file ppat.1003802.s017.doc]

|  | *U’’*(B’->B’’) | *r’’* | *a_mb’_*  (x10^-6^) | *Uis’’*(Muc’->Muc’’) | *r_m’’_* | *a_mmuc’’_*  (x10^-6^) |
| --- | --- | --- | --- | --- | --- | --- |
| **C*** | 4.3 x 10^-8^ | 2.47309513 | -3.7 | 1.72 x 10^-7^ | 2.3428444 | -3.24 |
| **D*** | 1 x 10^-8^ | 2.494764 | -3.7 | 1 x 10^-8^ | 2.32021774 | -2.99 |
| **E*** | 4.3 x 10^-8^ | 2.482068 | -3.7 |  |  |  |
