# Supplementary material for: The Genetic Basis of Escherichia coli Pathoadaptation to Macrophages
Source: PLoS Pathog. 2013 Dec 12;9(12):e1003802. doi: 10.1371/journal.ppat.1003802 (PMC3861542; doi:10.1371/journal.ppat.1003802)
Supplement: Text S1 — Includes Model of Clonal interference, Supplementary Materials and Methods and Supplementary References. (DOC) [file ppat.1003802.s018.doc]

**SUPPLEMENTARY TEXT S1**

**Model of clonal interference for the antagonistic interaction between bacteria and macrophages**

We assume a simple model for the interaction between bacteria and MΦ: , where *B* represents the number of bacteria along time, *r* their growth rate, *K* their carrying capacity and δ the death rate of macrophages. For the experimental conditions used *M*_Φ_=10^6^ and *B*(0)=10^6^. During a 24 hour period the infection dynamics expected under this model are similar to those obtained experimentally, with *r*=2.3 (per hour), *K*=10^8^ and δ=0.1 (per hour) (Fig. S1B). As in the experiments, every 24 hours bacterial population numbers are reduced to *B*(0) and macrophage numbers increased to *M*_Φ_. Having these initial conditions, we then assume that new mutants arise and are not stochastically lost at a given rate. More specifically, the ancestral clone can mutate to two new types of adapted clones: one by transposition (upstream of *yrfF*) and another by point mutation. These mutations can cause changes in both *r* and *a_m_*, i.e., the growth rate of mutant bacteria (*r_muc_*) can change and their ability to interact with macrophages (*a_mmuc_*) can also change. Given the experimental evidence in Figure 3, we assume that mucoid bacteria will exhibit an increased ability to escape macrophages *a_mmuc_* < *a_m_* but also a decreased growth rate *r_muc_* < *r*, due to the cost of producing exopolysaccarides. The following equations determine the evolution of a population where 3 new haplotypes can successfully emerge:

where *U* and *U_is_* are the rates of occurrence of successful beneficial SNPs and transpositions, respectively. Note that *U* is the spontaneous rate of mutation times the probability that such mutation is not lost by drift. **B** represents the number of bacteria with the ancestral genotype, **Muc** of the first mucoid genotype that emerges, **B’** of a derived genotype exhibiting a non-mucoid phenotype and **Muc’** of a derived mucoid genotype.

In this model of clonal interference we make the simplifying assumption that two traits, growth rate (*r*) of bacteria and their ability (*am*) to escape MΦ, are the most important for bacterial fitness in this environment. Both these traits can evolve, as it is evident from the emergence of new morphologies (in particular mucoid morphs) and phenotypic tests of the evolved clones (see Methods, Fig. 3, Fig. S7 and S8). Figure 5 shows simulations of adaptive dynamics over the period of the experiment (30 days), where the frequencies of mucoid (Muc) phenotypes are plotted and can be compared to those observed in the experiments. The solutions for the equations were obtained in Mathematica v8.0 (see full script in Protocol S1). These parameters were chosen because they depict the initial infection dynamics of the ancestral strain (Fig. S1) and its relation with the derived evolved clones (see Methods). We are able to find conditions that can reproduce the observed dynamics of morphs under a scenario of intense clonal interference and accumulation of multiple beneficial mutations (see Fig. S9). The model is able to reproduce the observed changes in frequency of the mucoid and non-mucoid phenotypes, if we assume that distinct beneficial mutations occur and change each of the two fitness traits in both morphs. Specifically, we assume that a successful beneficial mutation occurs, which produces the first mucoid morph. Such mutation is assumed to increase the ability of bacteria to escape MΦ, but also carry a cost in that it diminishes the growth rate (see Fig. S10 for the conditions under which such a mutation can invade). This mucoid morph can acquire further beneficial mutations, which can alter those traits values, such that a new derived mucoid haplotype can have a reduced cost of producing colanic acid and/or an increased ability to escape MΦ. Importantly, we also assume that clones with ancestral colony morphology can acquire beneficial mutations which increase their growth rate. Experimental support for this assumption is provided in the supplement (Fig. S11). Under these assumptions, and with the direct evidence that several distinct clones are segregating in the populations, complex dynamics are to be expected (Fig. 5 and Fig. S9).

It is important to note that we cannot exclude the possible occurrence of other forms of selection during the evolution experiment. Indeed it is known that even in simple abiotic environments adaptive diversification, involving frequency dependent selection can repeatedly evolve. It is generally difficult to distinguish this form of selection from simple clonal interference . However, if strong negative frequency dependent selection would have occurred in our lines, it would have led to the maintenance of distinct lineages along the evolutionary process. Such expectation seems inconsistent with the fixation of the IS insertion upstream of *yrfF* and the observed fixation of the mucoid phenotype in the majority of the lines.

**SUPPLEMENTARY MATERIALS AND METHODS**

**MIC determination**

Minimal inhibitory concentrations for aminoglycosides (gentamicin – 10 µg/ml, kanamycin – 30 µg/ml, amikacine – 30 µg/ml, tobramycin – 10 µg/ml, netilmycin – 10 µg/ml) and other type of antibiotics (nalidixic acid – 30 µg/ml, tetracycline - 30 µg/ml) were determined for the small and large colonies using antibiotic disc tests (LIOFILCHEM s.r.l., Italy) following manufacturer`s instructions.

**Bacterial survival inside** **MΦ**

To evaluate bacterial survival inside MΦ, a gentamicin protection assay was performed as previously described , with slight modifications. MΦ were seeded in 24-well tissue culture plate at approximately 2×10^5^ to 3×10^5^/ml and allowed to attach overnight. The cells were then washed, resuspended in fresh antibiotic-free RPMI medium, and activated with 2 μg of CpG-ODN 1826. After 24 h, the cells were washed from the remaining CpG-ODN, and fresh antibiotic-free RPMI medium was added. MΦ were infected with 5 × 10^6^ bacteria and centrifuged at (1000 rpm for 5 min). After 2 h of infection, the MΦ were washed from the extracellular bacteria, and fresh cell culture medium containing 100 μg of gentamicin/ml was added to kill the remaining extracellular bacteria. After incubation for an additional hour, the medium was removed, monolayers of MΦ were washed, and RPMI medium containing 20 μg of gentamicin/ml was added (0 h post-infection time point). To determine the number of intracellular bacteria at 0 h and 24 h of pos-tinfection, infected MΦ were washed twice with PBS, and 0.1% Triton-X was added for 30 min at 37°C in order to lyse the MΦ. The MΦ were then centrifuged (10000 rpm for 5 min), washed in PBS, and the overall number of bacteria was counted by plating them on LB agar plates. Survival inside the MΦ was estimated as the ratio between numbers of recovered bacteria at 0 h and 24 h.

**Bacterial growth in the presence versus absence of polyamines**

Bacterial cultures were grown in the presence and absence of spermidine (10 mM) or spermine (1 mM) as previously described , with slight modifications. Briefly, 15 μl of 10^-3^ bacterial culture (grown for 24 h in RPMI) was used to inoculate 135 μl RPMI media, covered in oil and grown at low speed agitation (5 s every 30 min). The carrying capacity (K) was measured using a Bioscreen C system (OD_600_). Relative growth was calculated as the ratio between K in the presence and absence of polyamines (see Fig S5).

**EPS quantification**

To quantify the total amount of exopolysaccharides (EPS), bacterial cultures were grown for 24 h in 1 ml RPMI media, heat-inactivated at 100 ^o^C for 15 min, centrifuged at 14000 rpm for 15 min and the supernatant collected for colorimetric assay to measure the binding of Congo red (adapted from Black and Yang ). A total of 450 µl supernatant was mixed with 50 µl of a dye (stock solution 150 µg/ml in deionized distilled water). All samples were vortexed briefly and incubated in the dark at room temperature for 30 min. The absorbances of the supernatants were measured at 490 nm in triplicate. Relative amount of EPS was estimated per bacterial cell by calculating CFUs by Flow Cytometry.

**Growth parameters of ancestral-looking bacteria**

We tested for changes in growth of 36 clones exhibiting an ancestral morphotype (4 replicates/clone) compared with the original ancestral clone (20 replicates). Growth rate measurements were obtained by following OD_600nm_ using a Bioscreen C system (Oy Growth Curves Ab). Cultures were done by seeding 150 μl RPMI MOI media with 10^4^ bacteria, covered with oil, and low speed agitation (5 s every 30min).

**Mutation frequency estimation and spontaneous *lon*::IS186 mutation rate estimation**

The clone M3_D19 was tested for increased mutagenesis, due to increased transposition, in comparison with the ancestral strain. A fluctuation test was performed to determine the number of D-cycloserine resistant mutants, a phenotype caused by a knock out of *cycA* gene, which can occur through IS insertions . Fluctuation assays were performed and the number of bacteria resistant to D-cycloserine (resistance can occur due to mutations or transpositions into the *cycA* gene) and to rifampicin (to which resistant mutants typically carry point mutation in *rpoB*, without involving transposition) were counted. D-cycloserine resistance assays were performed as previously described , with slight modifications. Briefly 50 tubes with 1 ml RPMI medium were inoculated with 5x10^4^ each which grew for 24 hours. 100 µl of different dilutions from each tube were then spread on M9 minimal medium (MM) agar plates supplemented with 0.2 % glucose and D-cycloserine at the final concentration of 0.04 mM. The frequency of mutagenesis was estimated as the number of mutants observed on D-cycloserine plates, over the total number of cells (measured by Flow cytometry). Clones were tested for increase in spontaneous single point mutation rate by plating 100 μl of an overnight culture of both ancestral and MUC_M3_D19 in LB supplemented with rifampicin at the end-concentration of 100 μg/mL.

To test for differences in the frequency of spontaneous mutation in the presence versus absence of MΦ we followed a previously described macrophage-induced mutagenesis assay , with slight modifications. Bacterial cultures of the ancestral strain were grown overnight (12 replicates for each treatment) and spread on furazolidone plates (1.25 µg/ml) . The frequency of mutagenesis was estimated as the number of nitrofuran-resistant mutants observed on furazolidone plates, over the total number of CFUs estimated in LB plates at appropriate dilutions. To determine the frequency of IS elements causing resistance to nitrofuran, the *nfsA* gene was amplified (using the primers in Table S2) from a random sample of nitrofuran-resistant clones. Insertion events were scored as increases in amplified band sizes. The presence of MΦ did not cause any significant difference in insertion frequency (with MΦ, IS frequency = 52% (51 out of 98 sampled clones), without MΦ the frequency was 59% (50 out of 85 sampled clones)).

To determine if the proportion of *lon*::IS186 spontaneous mutations in ANC clone was different in the presence versus absence of MΦ, we used a comparative PCR approach with primers IS186B and lonF, designed to detect IS186 inserted in the lon promoter (Table S2). Briefly, the ANC clone was grown three times in the presence and three times in the absence of MΦ (MOI 10^6^ to 10^6^) for 24 hours after which DNA was extracted. Amplifications with T22 clone (a clone with a stable IS186 insertion in *lon* gene) were used as a reference. We found the same average frequency of 2.7x10^-5^ (2SE=1x10^-5^) of spontaneous mutant clones when the ANC strain was grown in the presence and in the absence of the MΦ.

**In vitro infection of peritoneal macrophages**

8-10 weeks old C57BL/6J mice received an intraperitoneal dose of 2 mL thioglycollate (3%, Fluka). Three days later, mice were sacrificed by CO_2_ inhalation and macrophages were collected by washing the peritoneal cavity with 3 mL of PBS. Cells were plated for 3 hours in RPMI 1640 (Gibco) supplemented with 10% heat-inactivated FCS (Gibco), 2 mM L-glutamine (Invitrogen), 1 mM sodium pyruvate (Invitrogen), 10 mM HEPES (Invitrogen), 100 μg/ml streptomycin (Sigma; complete medium) and next, non-adherent cells were removed. Adherent cells (macrophages) were scraped, seeded in complete medium on 96-well plate (50 000 cells/well) and cultured overnight. On the following day, macrophages were left untreated or infected with MOI 0.01 of either ANC, CON (mixture of 6 clones independently evolved in the absence of macrophages) or MUC (mixture of 6 clones independently evolved in the presence of macrophages, MUC1 to MUC6). After adding *E. coli* to the macrophage culture, plates were centrifuged for 5 minutes at 150 g in room temperature and then kept for 4 hours. Next, media were collected, spun down (2 min, 150 g, room temperature) and stored frozen. TNF-α concentrations were measured in media collected from peritoneal macrophages infected with *E. coli* by ELISA (eBioscience) according to manufacturer instructions. No significant differences were found in the levels of TNF-α between the different strains of bacteria (Fig. S4).

**SUPPLEMENTARY REFERENCES**

1. Herron MD, Doebeli M (2013) Parallel evolutionary dynamics of adaptive diversification in Escherichia coli. PLoS Biol 11: e1001490.

2. Miskinyte M, Gordo I (2013) Increased survival of antibiotic-resistant Escherichia coli inside macrophages. Antimicrob Agents Chemother 57: 189-195.

3. Yohannes E, Thurber AE, Wilks JC, Tate DP, Slonczewski JL (2005) Polyamine stress at high pH in Escherichia coli K-12. BMC Microbiol 5: 59.

4. Black WP, Yang Z (2004) Myxococcus xanthus chemotaxis homologs DifD and DifG negatively regulate fibril polysaccharide production. J Bacteriol 186: 1001-1008.

5. Trindade S, Sousa A, Gordo I (2012) Antibiotic resistance and stress in the light of Fisher's model. Evolution 66: 3815-3824.

6. Feher T, Cseh B, Umenhoffer K, Karcagi I, Posfai G (2006) Characterization of cycA mutants of Escherichia coli. An assay for measuring in vivo mutation rates. Mutat Res 595: 184-190.

7. Schlosser-Silverman E, Elgrably-Weiss M, Rosenshine I, Kohen R, Altuvia S (2000) Characterization of Escherichia coli DNA lesions generated within J774 macrophages. J Bacteriol 182: 5225-5230.

8. Whiteway J, Koziarz P, Veall J, Sandhu N, Kumar P, et al. (1998) Oxygen-insensitive nitroreductases: analysis of the roles of nfsA and nfsB in development of resistance to 5-nitrofuran derivatives in Escherichia coli. J Bacteriol 180: 5529-5539.
